# Metal-free mechanochemical oxidations in Ertalyte^®^ jars

**DOI:** 10.3762/bjoc.15.172

**Published:** 2019-07-25

**Authors:** Andrea Porcheddu, Francesco Delogu, Lidia De Luca, Claudia Fattuoni, Evelina Colacino

**Affiliations:** 1 Dipartimento di Scienze Chimiche e Geologiche, Università degli Studi di Cagliari, Cittadella Universitaria, SS 554 bivio per Sestu, 09042 Monserrato (Ca), Italy; 2 Dipartimento di Ingegneria Meccanica, Chimica e dei Materiali, Università degli Studi di Cagliari, via Marengo 2, 09123 Cagliari, Italy; 3 Dipartimento di Chimica e Farmacia, Università degli Studi di Sassari, via Vienna 2, 07100-Sassari, Italy; 4 Université de Montpellier & Institut Charles Gerhardt de Montpellier (ICGM), UMR 5253 CNRS – UM – ENSCM, 8 Rue de l’Ecole Normale, 34296 Montpellier, Cedex 5, France

**Keywords:** AZADO, Ertalyte^®^, green chemistry, mechanochemistry, NaOCl·5H_2_O, selective oxidation, TEMPO

## Abstract

Aimed at eliminating or at least significantly reducing the use of solvents, sodium hypochlorite pentahydrate crystals (NaOCl·5H_2_O) in the presence of a catalytic amount of a nitrosyl radical (TEMPO or AZADO) have been successfully used to induce mechanochemical oxidative processes on several structurally different primary and secondary alcohols. The proposed redox process is safe, inexpensive and performing effectively, especially on the macroscale. Herein, an Ertalyte^®^ jar has been successfully used, for the first time, in a mechanochemical process.

## Introduction

The conversion of primary and secondary alcohols to the corresponding carbonyl compounds (aldehydes and ketones, respectively) is of such importance in organic chemistry that it finds very few parallels in other synthetic organic processes [1–2]. These transformations can be achieved by using a wide range of oxidizing reagents [3], but most of them are difficult-to-handle and suffer from waste problems due to large amounts of byproducts, thus decreasing the atom efficiency [4–5]. The discovery of (2,2,6,6-tetramethylpiperidin-1-yl)oxyl, commonly known as TEMPO by Lebedev and Kazarnowskii in 1960 has been hailed as a significant breakthrough in the field of redox reactions, allowing the fast and selective oxidation of alcohols to the related carbonyl compounds under very mild conditions [6–7]. Initially used in a stoichiometric amount [8], over the last 20 years it has been exploited successfully in catalytic quantities in combination with other oxidants [9]. A diverse range of co-oxidant agents (*N*-chlorosuccinimide, NaOCl, Oxone^®^, PhIO, PhICl_2_, PhI(OAc)_2_, I_2_, CAN, etc.) has been intensively investigated with varying results in terms of yields, chemical selectivity, and environmental sustainability [10–18]. All oxidation procedures have their advantages and their flaws, so the search for efficient, selective, high-yielding, environmentally benign methods and atom-economical processes continues to be a pivotal challenge for chemists [19]. Stahl and many other researchers worked in this direction achieving noteworthy results by using air/oxygen as an oxidizing agent in the presence of a suitable metal complex [20–25]. However, even these recent methods suffer from serious drawbacks, such as the use of precious metals often combined with sophisticated organic ligands, which makes them expensive, especially if implemented on an industrial scale. In addition, increasingly restrictive legislation against residual metals in manufactured goods and active ingredients stimulates the ongoing search for new metal-free solutions to the problem making this challenge even topical [26–27]. Based on the considerations mentioned above, we focused on an alternative strategy to activate the oxidation process. In particular, in this study, we used sodium hypochlorite pentahydrate (NaOCl·5H_2_O) in the presence of a catalytic amount of a nitrosyl radical (TEMPO or AZADO) to induce mechanochemical oxidation reactions on suitably selected primary and secondary alcohols. Performed in a high-energy ball mill and with the unprecedented utilization of Ertalyte^®^ jars, the mechanical activation allows obtaining the oxidized products from a broad spectrum of initial substrates. We show that the proposed mechanochemical method is definitely safe, performing effectively and inexpensive, thus providing an interesting synthetic route that can be scaled up to pilot and industrial levels.

## Results and Discussion

Since the most commonly employed oxidizing agents are solid reagents, we decided to develop an efficient and eco-friendly process for the selective oxidation of alcohols to the corresponding aldehydes/ketones based on a mechanochemical activation [28]. In comparison to solution-based techniques, ball-milling procedures provide an ideal solution for overcoming many of the drawbacks described above, due to the simplicity of use, shorter reaction times, large-scale production, low cost and sustainability of this methodology [28–39]. In addition, impact forces, that are generated by ball-milling media involve a very minimal fraction of reactive material mimicking the ideal behavior/trend of highly diluted reactive systems. This peculiar aspect of mechanochemical reactions, especially in redox processes conducted in no-metal reactors, could prevent excessive heating of the jar, avoid the decomposition of starting materials and therefore, limit the formation of byproducts [40]. Following our interest in mechanochemistry and the design of new cost-effective oxidation procedures, we have tried to combine both topics to the best [41–49]. In particular, we were mainly interested in developing a general, selective and versatile alcohol-to-aldehyde/ketone oxidative protocol applied to primary and secondary alcohols by using an oxidizing agent as cheap and eco-friendly as possible.

In order to optimize all the experimental conditions, we fine-tuned the reaction by using 3-phenyl-1-propanol as a model reagent and *N*-chlorosuccinimide (NCS) as an oxidizing agent. NCS is one of the most widely used co-oxidizing reagents in homogeneous-phase TEMPO-assisted oxidation reactions and, we have gained valuable experience in handling this reagent in several mechanochemical applications [45–46].

**Scheme 1:**
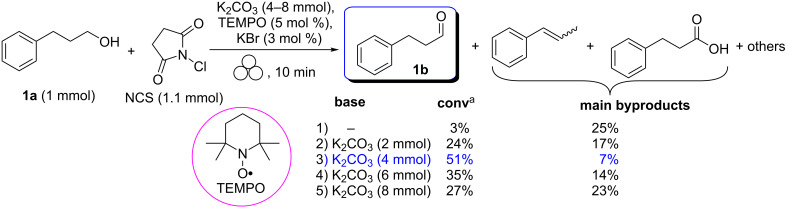
Oxidation of 3-pheny-1-propanol (**1a**) with *N*-chlorosuccinimide (NCS) in the presence of (2,2,6,6-tetramethylpiperidin-1-yl)oxyl (TEMPO) under mechanical activation conditions [50]. ^a^Percentages of conversion were calculated by GC–MS using an internal reference standard.

*N*-Chlorosuccinimide (1.1 mmol) and 3-pheny-1-propanol (1.0 mmol) were milled together in the presence of TEMPO (5 mol %), K_2_CO_3_ (4.0 mmol) and KBr (3.0 mol %) for 10 minutes in a zirconia jar containing 5 balls (5 mm Ø) of the same material (Scheme 1).

For all the experiments, we never observed a complete conversion of the alcohol into the aldehyde. Moreover, the first experimental results showed the key role of the base in the conversion of an alcohol into an aldehyde: it fails in the absence of K_2_CO_3_, reaches a maximum of 51% with 4 equivalents and decreases (27%) for higher amounts (Scheme 1). This is mainly due to the fact that the concentration of the active oxidizing agent, HOCl is strongly dependent on the amount and strength of the base used in the grinding mixture [51].

**Scheme 2:**
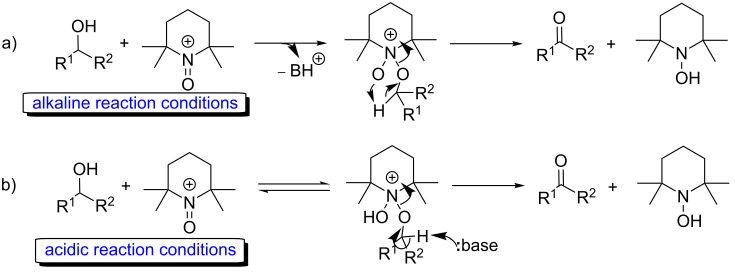
Hypothesized pathways for the TEMPO-assisted oxidation of alcohols in a) basic or b) acidic reaction conditions.

Two different mechanisms have been postulated for similar reactions in homogeneous phase: one occurs under acidic conditions, while the other works better in a basic medium through a cyclic dipolar mechanism (Scheme 2) [52–53]. On the contrary, under ball-milling conditions, it is possible to hypothesize that only the cyclic dipolar mechanism, which operates mostly in basic conditions, allowed to gain access to the desired aldehyde.

Interestingly, the formation of a significant amount of over-oxidation [54] and elimination byproducts was observed for 3-pheny-1-propanol (**1a**) when increasing the reaction time (up to 20 min, Scheme 1). In contrast, comparable results were obtained when the milling time was reduced to 3 minutes, leading to an alcohol/aldehyde ratio very similar to that one obtained after ten minutes. Any attempt to improve this conversion by varying other parameters such as the number (up to 15 balls, 5 mm Ø) and the diameter of balls (from 3 up to 10 mm Ø), or using a different base (KHCO_3_ or Na_2_CO_3_) turned out to be unsuccessful.

**Scheme 3:**
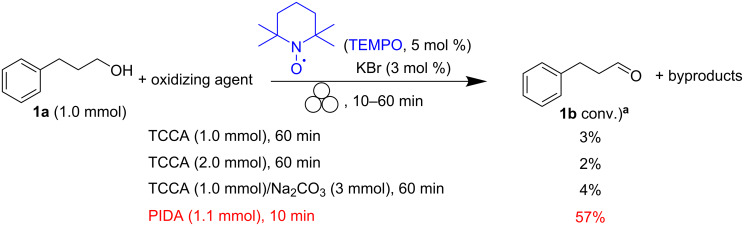
TEMPO-assisted oxidation of 3-pheny-1-propanol (**1a**) under mechanical activation conditions. ^a^Percentages of conversion were calculated by GC–MS using an internal reference standard.

The use of other solid oxidants such as trichloroisocyanuric acid (TCCA) did not bring any advantage to the process (Scheme 3), and the aldehyde was only detected in negligible amounts (GC–MS analyses). The (diacethoxyiodo)benzene acid (PIDA) allowed to further improve the alcohol-to-aldehyde conversion by a few percentage points (57%), but the formation of 2 equivalents of acetic acid makes it unsuitable for a mechanochemical process [55]. Also, Oxone^®^ and NH_2_CONH_2_·H_2_O_2_ appeared to fail in the oxidation of 3-phenyl-1-propanol (**1a**) to the corresponding aldehyde.

Subsequently, we turned our attention to sodium hypochlorite (NaOCl), an inexpensive and widely used oxidizing reagent also applied as a disinfectant and household bleaching agent, usually sold as a 3–6% solution in water. Commercially available NaOCl is highly basic (pH ≈ 12.7) that dramatically slows down the oxidation process, and NaHCO_3_ has to be added to both maximize the concentration of the active oxidizing agent HOCl, and to absorb bleach [56]. The addition of a catalytic amount of KBr promotes the in situ generation of HOBr, which is a stronger oxidant than HOCl [57].

The results improved remarkably by using as oxidant a 6% aqueous solution of NaOCl (1.14 mL, 1.1 mmol) adsorbed on NaHCO_3_ (6.5 g) in the presence of a catalytic amount of TEMPO (5.0 mol %) and KBr (3.0 mol %) (Table 1, entry 6). Within 20 minutes, the alcohol was completely and selectively oxidized into the corresponding aldehyde (as assessed by GC–MS analyses). The use of NaCl, alone or in combination with NaHCO_3_, as an adsorbent [58] (Table 1, entries 1–5, 7) or bases (Na_2_CO_3_, Table 1, entries 7 and 8) other than NaHCO_3_ significantly reduces the alcohol-to-aldehyde conversions.

**Table 1:** Oxidation of 3-phenyl-1-propanol (**1a**) with aqueous NaOCl (6%, bleach) under mechanical activation conditions [50].

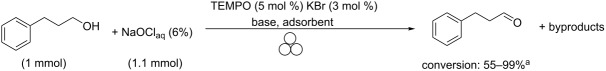					

Entry	Base	NaCl (g)^a^	Time (min)	Aldehyde (%)^b^	Byproducts (%)^b^

1	NaHCO_3_ (2.0 g)	3.5	15	73	16
2	NaHCO_3_ (2.0 g)	3.0	15	77	22
3	NaHCO_3_ (1.0 g)	5.0	15	78	8
4	NaHCO_3_ (1.0 g)	5.0	30	78	6
5	NaHCO_3_ (2.5 g)	2.5	15	88	4
**6**	**NaHCO_3_ (6.5 g)**	**–**	**22**	**>99**	**–**
7	Na_2_CO_3_ (2.0 g)	3	22	61	37
8	Na_2_CO_3_ (6.0 g)	–	22	55	41

^a^NaCl was used as an adsorbent [58] in combination with NaHCO_3_ or Na_2_CO_3_. ^b^Percentages of conversion were calculated by GC using an internal reference standard.

Based on these preliminary results, we decided to replace aqueous NaOCl (bleach) with Ca(OCl)_2_ that has been reported previously as a valid alternative to NaOCl aqueous solutions for mechanochemical chlorination reactions of hydantoins [41,59]. However, also using this oxidant, we observed low conversions (31%) and the formation of significant amounts of byproducts, mainly halides and olefins (elimination byproducts). The use of liquid-assisted grinding (LAG) procedures [60–62] by adding small quantities of water (250 μL) improved the performance of the reaction (alcohol-to-aldehyde conversion: 41%), but also raised the percentage of elimination products (38%). Solid NaOCl·5H_2_O, which has been discovered over a century ago, represented a turning point in our search for a suitable reagent, avoiding some of the previously described shortcomings. As of 2013, this reagent is commercially available [63], inexpensive and sufficiently stable and safe for potential applications in mechanochemistry (Figure S1a, Supporting Information File 1) [64–67].

Once the most promising oxidant was identified, NaOCl·5H_2_O (1.1 mmol), 3-phenyl-1-propanol (1.0 mmol), NaHCO_3_ (2.2 mmol), and KBr (3 mol %) were milled together in the presence of TEMPO (5 mol %) for 20 minutes in a zirconia jar containing 6 balls (5 mm Ø) of the same material. NaHCO_3_ plays the double role of base and adsorbent for liquid alcohols (Table 2, entry 1). The first results were promising and showed a good alcohol-to-aldehyde conversion (75%). We have also used a Teflon jar, but we observed lower conversions (<50%). In addition, the reproducibility of data was often poor. In our ongoing efforts to develop mechanochemical reactions in jars manufactured from thermoplastic materials as alternatives to Teflon, having high mechanical resistance, rigidity, and hardness, we were pleased to find that Ertalyte^®^ displayed an excellent performance in the mechanical process. All other parameters being equal, the conversion efficiency improved significantly by switching to an Ertalyte^®^ jar (86%) [68] which could be further enhanced (93%) by slightly increasing the amount of the oxidant agent (1.5 equiv). Ertalyte^®^ jars (Figure S1b, in Supporting Information File 1) are composed of polyethylene terephthalate (PET-P) and characterized by wear- and abrasion-, chemical and moderate acid resistance, with a low coefficient of friction and FDA approved [69].

**Table 2:** Oxidation of 3-phenyl-1-propanol (**1a**) with NaOCl·5H_2_O crystals under mechanical activation conditions using ZrO_2_ or Ertalyte^®^ jars. Optimization of the reaction conditions.

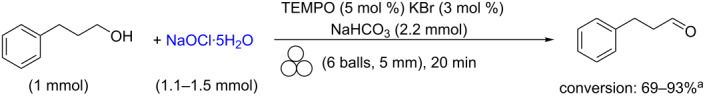					

Entry	NaOCl·5H_2_O (mmol)	Jar	Aldehyde (%)^a^	Alcohol (%)^a^	Byproducts (%)^a^

1	1.1	Zirconia	75	19	6
2^b^	1.1	Zirconia	70	11	19
3	1.1	Ertalyte^®^	86	9	5
**4**	**1.5**	**Ertalyte^®^**	**93**	**5**	**2**
5^c^	1.5	Ertalyte^®^	69	27	4

^a^Percentages of conversion were calculated by GC using an internal reference standard. ^b^The reaction time was extended to 40 min. ^c^The amount of TEMPO was decreased from 5 to 3 mol %.

**Scheme 4:**
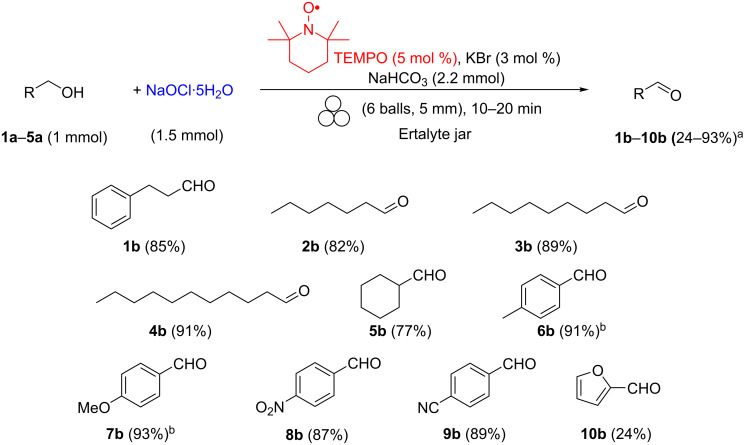
Scope of primary alcohol oxidation under mechanical activation conditions. ^a^All yields refer to isolated pure products. ^b^The compound was prepared according to the general procedure A (see Supporting Information File 1) without adding TEMPO catalyst, and the reaction was completed within ten minutes.

In the absence of TEMPO, the oxidation reaction did not work anymore, while in the absence of KBr, the conversion rate was considerably reduced. The use of bases other than NaHCO_3_ (Na_2_CO_3_ or sodium citrate) resulted in low alcohol-to-aldehyde conversions (<40%) and promoted, conversely, the formation of significant amounts of olefins (>25%) resulting from halide elimination. Any attempt to lower the amount of the nitrosyl catalyst resulted in a poor conversion (69%, Table 2, entry 5). Once the reaction conditions were fine-tuned, this procedure was applied to a range of alcohols to assess the scope of the reaction. The results are shown in Scheme 4.

Aliphatic alcohols **1a**–**5a** with carbon chains of different length were oxidized to give the corresponding aldehydes **1b**–**5b** in good yields and no carboxylic acid derivatives were observed in any sample. Similar results were obtained for alcohols containing an aliphatic carbon ring in their backbone, such as cyclohexylmethanol (**5a**). Interestingly, the oxidation reaction of benzylic alcohols proceeded smoothly to completeness in about 10 minutes even without need for TEMPO.

The results changed significantly with benzylic alcohols decorated with an electron-withdrawing group in the aromatic ring such as 4-nitrobenzylalcohol (and 4-cyanobenzylalcohol), which required 5 mol % of TEMPO to be oxidized.

**Scheme 5:**
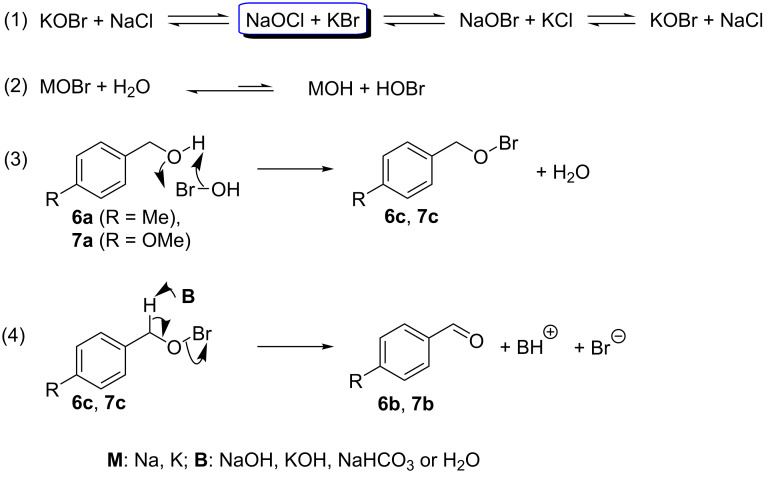
Proposed mechanism for the oxidation of benzylic alcohols **6a** and **7a** under mechanochemical conditions and in the presence of KBr.

Based on these experimental results, we hypothesize that the reaction proceeded by a mechanism different from the classical solution-based TEMPO-assisted oxidation of alcohols, as illustrated in Scheme 5 [65]. In the first step of the reaction, potassium bromide, used as a co-catalyst, generates in situ sodium hypobromite, a more favorable oxidizing reagent than sodium hypochlorite. Ion metathesis due to the presence of KCl may lead to KOBr (Scheme 5, reaction 1). Subsequently, the species MOBr (M = Na, K) reacts with water to form HOBr, which is the active oxidizing agent (Scheme 5, reaction 2). Once the oxidizing agent formed, it reacts with the benzylic alcohols **6a** or **7a** to afford the corresponding intermediate benzyl hypobromites **6c** or **7c** (Scheme 5, reaction 3). In the final step, the base deprotonates the acidic benzylic proton leading to the corresponding benzaldehyde **6b** or **7b** (Scheme 5, reaction 4).

**Scheme 6:**
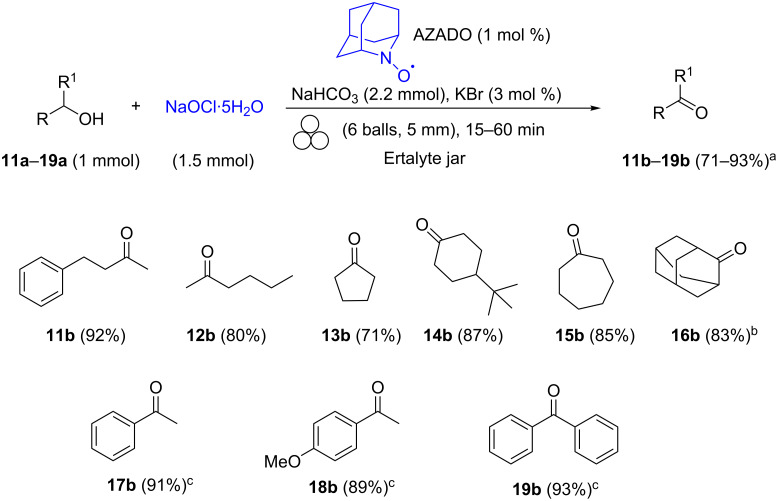
Scope of secondary alcohols in the oxidation under mechanical activation conditions. ^a^All yields refer to isolated pure products. ^b^2-Aza-adamantane**-***N*-oxyl (AZADO, 2 mol %), 60 min. ^c^Oxidation carried out without AZADO catalyst, 15 min. The title compound was prepared according to the general procedure B (see Supporting Information File 1).

The oxidation of furfuryl alcohol gave furfural (**10b**), but in low and irreproducible yields together with significant amounts of byproducts. As expected, the reaction with conjugated alcohols (cinnamic alcohol, propargyl alcohol, etc.) was less successful due to the competing chlorination of the multiple bonds. Prompted by these findings, we further explored the efficacy of the protocol with a variety of secondary alcohols. Unfortunately, the oxidation reaction tested on 4-phenyl-2-butanol proceeded with low conversion yields (45%). An increase in both the amount of the oxidant (2 equiv) and the nitrosyl radical (10 mol %), as well as longer reaction times (up to 1.5 h), did not lead to any significant improvement. However, we were pleased to find that the less hindered 2-aza-adamantane-*N*-oxyl (AZADO) was more effective than TEMPO in terms of conversion and yield with the model alcohol substrate, promoting an almost quantitative conversion of 4-phenyl-2-butanol into benzylacetone in only 30 min (Scheme 6, ketone **11b**). This protocol was successfully extended to other secondary alcohols to afford the corresponding ketones **11b**–**19b** in high conversions and yields.

The oxidation of sterically hindered secondary alcohols such as adamantan-2-ol (Scheme 4, alcohol **16a**) required doubling of the quantity of the nitrosyl catalyst (AZADO, 2 mol %) and longer reaction times (from 30 to 60 min) to achieve completion. Another useful feature of this protocol can be seen in the case of secondary benzyl alcohols, where the oxidation reaction to the corresponding ketones proceeded smoothly even without the necessity to use the nitrosyl catalyst (Scheme 6, ketones **17b**–**19b**). With all benzylic alcohols examined, the GC–MS analyses showed that the reactions were nearly complete in about 15 minutes, ketones **17b**–**19b** being isolated in high yields and purities. Finally, we investigated if this protocol could be potentially implemented on a larger scale. Pleasingly, we were able to scale-up the oxidation of **1a** and **11a** from a 1 mmol up to a 10 mmol scale without any significant drop in terms of purity and yield, thus confirming the method’s potential adaptability to industrial settings.

**Scheme 7:**
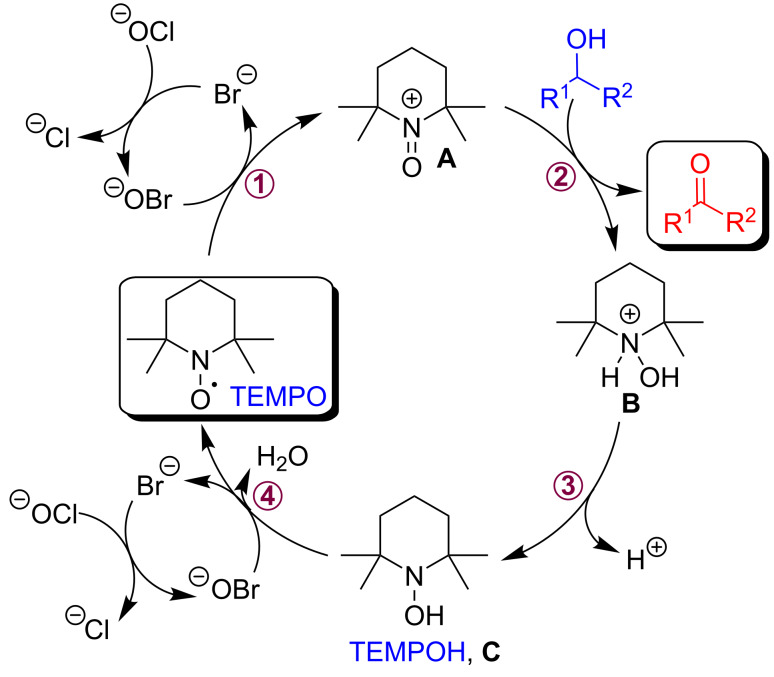
Possible mechanism for the TEMPO-mediated oxidation of primary and secondary alcohols by using NaOCl·5H_2_O and catalytic KBr.

The proposed mechanism for the TEMPO-based oxidative conversion of primary and secondary alcohols to the corresponding carbonyl compounds is described in Scheme 7 and shares similarities with that postulated in other previous studies in solution.

In general, NaOCl works as a co-oxidant agent, and in the catalytic cycle reacts with KBr generating in situ ^−^OBr, a stronger oxidizing species. Subsequently, a catalytic amount of ^−^OBr oxidizes the TEMPO radical to the *N*-oxo-ammonium ion **A**. The latter in turn rapidly oxidizes the alcohol to the corresponding carbonyl compound and gives the reduced form of TEMPO, the hydroxylamine **C**, TEMPOH. Then hydroxylamine **C** is reoxidized by ^−^OBr regenerating the starting TEMPO radical or directly the *N*-oxo-ammonium species **A**, thus closing the catalytic cycle (Scheme 7).

## Conclusion

The conversion of primary and secondary alcohols to aldehydes and ketones, respectively, is one of the most important reactions in the panorama of organic chemistry. Although the literature describes a plethora of reagents and methodologies, most of them use toxic/harmful reagents that often cause serious environmental and public health concerns. Crystalline sodium hypochlorite (NaOCl·5H_2_O) in the presence of a catalytic amount of a nitrosyl radical allowed developing a redox process without using any metal catalyst. With the aim to eliminate or at least reduce the use of solvents, NaOCl·5H_2_O, among all the oxidants tested, was the one that best fitted with a general mechanochemical oxidative process of alcohols in Ertalyte^®^ jars. The latter material never has been explored before in any of the mechanochemical transformations described in the literature and produced outperforming results compared to those obtained in zirconium oxide jars.

## Supporting Information

File 1 Experimental procedures, characterization of new compounds and copies of ^1^H and ^13^C NMR spectra.
