# Supplementary material for: Metal-free mechanochemical oxidations in Ertalyte® jars
Source: Beilstein J Org Chem. 2019 Jul 25;15:1786–94. doi: 10.3762/bjoc.15.172 (PMC6664414; doi:10.3762/bjoc.15.172)
Supplement: File 1 — Experimental procedures, characterization of new compounds and copies of 1H and 13C NMR spectra. [file Beilstein_J_Org_Chem-15-1786-s001.pdf]

BEILSTEIN JOURNAL OF ORGANIC CHEMISTRY

Supporting Information

for

Metal-free mechanochemical oxidations in Ertalyte® jars

Andrea Porcheddu, Francesco Delogu, Lidia De Luca, Claudia Fattuoni
and Evelina Colacino

Beilstein J. Org. Chem. **2019,** *15,* 1786–1794. doi:10.3762/bjoc.15.172

Experimental procedures, characterization of new compounds and copies of ^{1}H and ^{13}C NMR spectra

License and Terms: This is a supporting information file under the terms of the Creative Commons Attribution License (http://creativecommons.org/licenses/by/4.0). Please note that the reuse, redistribution and reproduction in particular requires that the authors and source are credited.
The license is subject to the *Beilstein Journal of Organic Chemistry* terms and conditions: (https://www.beilstein-journals.org/bjoc)

Table of contents

1. General methods and materials S2

2. Figure S1a and Figure S1b S3

3. General procedure for the oxidation of primary alcohols **1a**–**10a** (procedure A) S4

4. General procedure for the oxidation of secondary alcohols **11a**–**19a** (procedure B) S5

5. ^{1}H and ^{13}C NMR data for carbonyl compounds **1b–19b** S6

6. ^{1}H and ^{13}C NMR spectra for carbonyl compounds **1b**–**19b** S10

1. General methods and materials

Commercially available reagents were purchased from Acros, Aldrich, Strem Chemicals, Alfa-Aesar, TCI Europe and used as received. Sodium hypochlorite pentahydrate ($NaOCl \cdot 5H_2O$, CAS [10022-70-5] is commercially available from TCI chemicals (Product reference S0939). The solvents were purchased from Aldrich or VWR International in sealed bottles over molecular sieves. Flash column chromatography was performed with EcoChrome™ MP Silica gel 60A, particle size 0.040–0.063 mm (230–400 mesh). All reactions were monitored by thin layer chromatography (TLC) performed on glass-backed silica gel 60 F254, 0.2 mm plates (Merck), and compounds were visualized under UV light (254 nm) or using cerium ammonium molybdate solution with subsequent heating. The eluents were technical grade and distilled prior to use. Unless otherwise stated, all mechanochemical reactions were carried out in a FTS-1000 Shaker Mill® apparatus (Form-Tech Scientific, Canada), using homemade Ertalyte® milling jars. The Ertalyte® jar volume was 15 mL (40.6 g), the balls (6 × 5 mm Ø, total weight of balls 2.34 g) were made of zirconia, the milling rate equal to 1800 rpm and the milling time up to 60 min. Mechanochemical reactions on macro scale were performed in a 25 mL Ertalyte® milling jar (72.1 g) containing 45 balls (5 mm Ø, total weight of balls 21.33 g) of zirconia. ^{1}H and ^{13}C NMR spectra were recorded on a Bruker Avance III HD 600 NMR spectrometer (600 MHz, 14.09 tesla) at 25 °C and were calibrated using trimethylsilane (TMS). Proton chemical shifts are expressed in parts per million (ppm, δ scale) and are referred to the residual hydrogen in the solvent ($CHCl_3$, 7.27 ppm). Data are represented as follows: chemical shift, multiplicity (s = singlet, d = doublet, t = triplet, q = quartet, m = multiplet and/or multiple resonances, br s = broad singlet), coupling constant (*J*) in hertz *(Hz)* and integration. Carbon chemical shifts are expressed in parts per million (ppm, δ scale) and are referenced to the carbon resonances of the NMR solvent ($CDCl_3$, δ 77.0 ppm or δ DMSO-d_6 δ 39.5 ppm). Deuterated NMR solvents were obtained from Aldrich. High-resolution mass spectra (HRMS) were recorded using an Electrospray Ionization (ESI) spectrometer. Analysis of reaction mixture was determined by GC–MS (GC Agilent 6850, MS Agilent 5973) and equipped with a HP5 capillary column (30 m length and 0.20 mm diameter, 0.11 film thickness). GC oven temperature was programmed from 80 °C to 250 °C at the rate of 10 °C/min. Helium was used as the carrier gas. Temperatures of injection port and FID were kept constant at 300 °C. Retention times of different compounds were determined by injecting pure samples under identical conditions. For quantification, *n*-dodecane was used as an internal standard for GC–MS analysis. Melting points were determined in an open capillary on a Büchi melting point apparatus and are uncorrected. All the experiments were carried out in duplicate to ensure reproducibility of the

experimental data. Yields refer to pure isolated materials. Spectroscopic data were in agreement with those of the commercially available reference compounds.

2. Figure S1a and Figure S1b

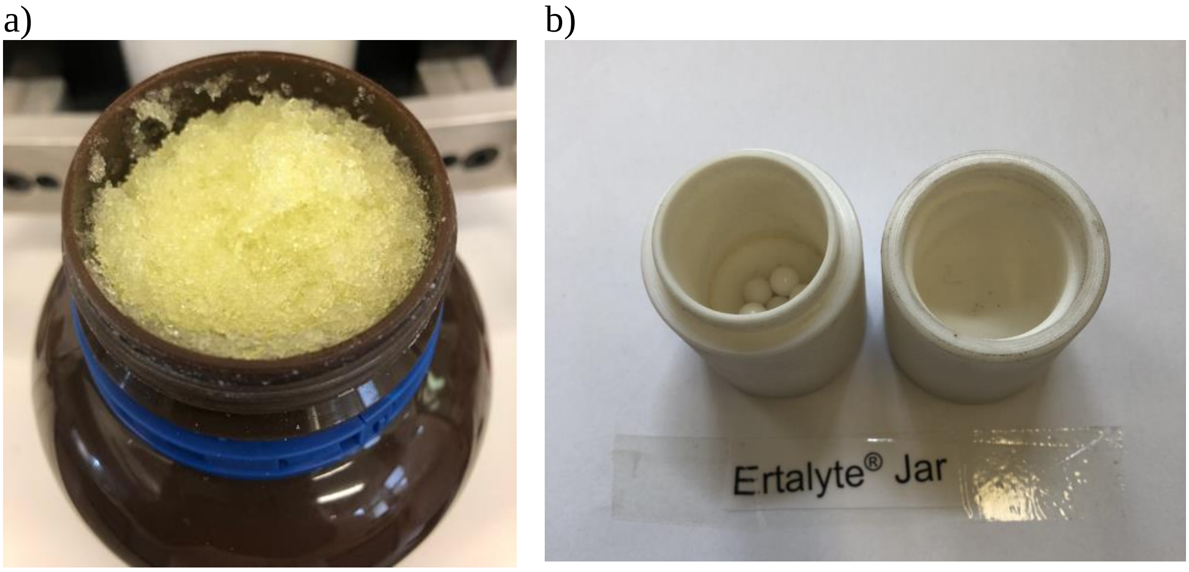

Figure S1: a) Commercial sodium hypochlorite pentahydrate crystals ($NaOCl{\cdot}5H_2O$); b) homemade Ertalyte® milling jar.

3. General procedure for the oxidation of primary alcohols 1a–10b (procedure A)

$NaOCl·5H_2O$ (247 mg, 1.5 mmol), $NaHCO_3$ (185 mg, 2.2 mmol), and KBr (3.6 mg, 0.03 mmol, 3 mol %) were placed in an Ertalyte® jar (15 mL, 40.6 g) equipped with six zirconia balls (5 mm Ø). The jar was ball-milled at 1800 rpm (30 Hz) for 1 min. Following this initial grinding period, primary alcohol **1a–10a** (1.0 mmol), and 2,2,6,6-tetramethylpiperidine-1-oxyl (TEMPO, 7.8 mg, 0.05 mmol, 5 mol %), were added and the reaction mixture was subjected to further grinding at 30 Hz for 20 minutes. The progress of the reaction was monitored by GC–MS analysis and TLC analysis (heptane/AcOEt 9:1, v/v) of an aliquot of crude. The milling was stopped, Na_2SO_3 (189 mg, 1.5 mmol) added to the jar, and milling continued at 30 Hz for further 3 minutes. Then, AcOEt (2 × 1.5 mL) was added into the jar and the crude was transferred into a round-bottomed flask together with silica gel (350 mg). The combined organic layers were concentrated in vacuo. The resulting residue was purified through a short column on silica gel with ethyl acetate/hexane 1:9 (v/v) as the eluent to afford the corresponding aldehydes **1b–10b**.

4. General procedure for the oxidation of secondary alcohols 11a–19a (procedure B)

$NaOCl\cdot5H_2O$ (247 mg 1.5 mmol), $NaHCO_3$ (185 mg, 2.2 mmol), and KBr (3.6 mg, 0.03 mmol, 3 mol %) were placed in an Ertalyte® jar (15 mL, 41.2 g) equipped with six zirconia balls (5 mm Ø). The jar was ball-milled at 1800 rpm for 1 min. Following this initial grinding period, secondary alcohol **11a–19a** (1.0 mmol), and 2-aza-adamantane-*N*-oxyl (AZADO, 1.6 mg, 0.01 mmol, 1 mol %), were added and the reaction mixture was milled at 30 Hz for further 30 minutes. The progress of the reaction was monitored by TLC (heptane/AcOEt 9:1, v/v) and GC–MS analysis of an aliquot of the crude. The milling was stopped, Na_2SO_3 (189 mg, 1.5 mmol) added to the jar. Then, milling was continued at 30 Hz for further 3 minutes. AcOEt (2 × 1.5 mL) was added into the jar and the crude was transferred to a round-bottomed flask together with silica gel (350 mg). The combined organic layers were concentrated in vacuo. The resulting residue was purified through a short column on silica gel with ethyl acetate/hexane 1:9 (v/v) as the eluent to yield the target ketones **11b–19b**.

5. ^{1}H and ^{13}C NMR data for carbonyls compounds 1b–19b

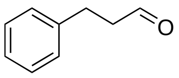

1b

3-Phenylpropanal (**1b**, 114 mg, 85%), CAS Number 104-53-0. The title compound was prepared according to the general procedure A. **^{1}H NMR** (600 MHz, $CDCl_3$) δ (ppm): 9.85 (s, 1H), 7.33 (t, *J* = 7.5 Hz, 2H), 7.24 (dd, *J* = 11.6, 7.2 Hz, 3H), 2.99 (t, *J* = 7.6 Hz, 2H), 2.81 (td, *J* = 7.6, 1.3 Hz, 2H). **^{13}C NMR** (151 MHz, $CDCl_3$) δ (ppm): 201.7, 140.4, 128.7, 128.4, 126.4, 45.4, 28.2.

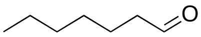

2b

1-Heptanal (**2b**, 94 mg, 82%), CAS Number 111-71-7. The title compound was prepared according to the general procedure A. **^{1}H NMR** (600 MHz, $CDCl_3$) δ (ppm): 9.78 (t, *J* = 1.9 Hz, 1H), 2.43 (td, *J* = 7.4, 1.9 Hz, 2H), 1.64 (p, *J* = 7.3 Hz, 2H), 1.39–1.26 (m, 6H), 0.90 (t, *J* = 7.0 Hz, 3H). **^{13}C NMR** (151 MHz, $CDCl_3$) δ (ppm): 202.9, 43.90, 31.5, 28.8, 22.4, 22.0, 14.0.

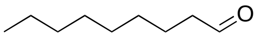

3b

1-Nonanal (**3b**, 127 mg, 89%), CAS Number 124-19-6. The title compound was prepared according to the general procedure A. **^{1}H NMR** (600 MHz, $CDCl_3$) δ (ppm): 9.76 (t, *J* = 1.9 Hz, 1H), 2.42 (td, *J* = 7.4, 1.9 Hz, 2H), 1.69–1.58 (m, 2H), 1.35–1.23 (m, 10H), 0.88 (t, *J* = 7.0, 3H). **^{13}C NMR** (151 MHz, $CDCl_3$) δ (ppm): 203.0, 43.9, 31.8, 29.3, 29.2, 29.1, 22.6, 22.1, 14.1.

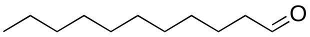

4b

1-Undecanal (**4b**, 155 mg, 91%), CAS Number 112-44-7. The title compound was prepared according to the general procedure A. **^{1}H NMR** (600 MHz, $CDCl_3$) δ (ppm): 9.78 (t, *J* = 1.9 Hz, 1H), 2.43 (td, *J* = 7.4, 1.9 Hz, 2H), 1.67–1.62 (m, 2H), 1.36-1.29 (m, 14H), 0.89 (d, *J* = 7.2 Hz, 3H).**^{13}C NMR** (151 MHz, $CDCl_3$) δ (ppm): 202.9, 43.9, 31.9, 29.5, 29.4, 29.3, 29.3, 29.2, 22.7, 22.1, 14.1.

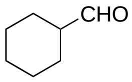

5b

Cyclohexanecarbaldehyde (**5b**, 87 mg, 77%), CAS Number 2043-61-0. The title compound was prepared according to the general procedure A. **^{1}H NMR** (600 MHz, $CDCl_3$) δ (ppm): 9.62 (d, *J* = 1.1 Hz, 1H), 2.26-2.21 (m, 1H), 1.92–1.87 (m, 2H), 1.75-1.64 (m, 2H), 1.68–1.59 (m, 1H), 1.38-1.30 (m, 4H), 1.28-1.21 (m, 1H).

^{13}**C NMR** (151 MHz, $CDCl_3$) δ (ppm): 205.0, 50.0, 26.0, 25.9, 25.0.

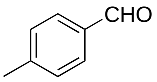

6b

4-Methylbenzaldehyde (**6b**, 109 mg, 91%), CAS Number 104-87-0. The title compound was prepared according to the general procedure A without adding TEMPO catalyst. ^{1}**H NMR** (600 MHz, $CDCl_3$) δ (ppm): 9.98 (s, 1H), 7.79 (d, *J* = 8.1 Hz, 2H), 7.34 (d, *J* = 8.1 Hz, 2H), 2.45 (s, 3H). ^{13}**C NMR** (151 MHz, $CDCl_3$) δ (ppm): 192.0, 145.5, 134.2, 129.8, 129.7, 21.9.

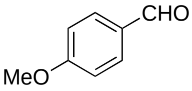

7b

4-Methoxybenzaldehyde (**7b**, 127 mg, 93%), CAS Number 123-11-5. The title compound was prepared according to the general procedure A without adding TEMPO catalyst. ^{1}**H NMR** (600 MHz, $CDCl_3$) δ (ppm): 9.89 (d, *J* = 1.8 Hz, 1H), 7.84 (d, *J* = 8.8 Hz, 2H), 7.01 (d, *J* = 8.8 Hz, 2H), 3.89 (s, 3H). ^{13}**C NMR** (151 MHz, $CDCl_3$) δ (ppm): 190.8, 164.6, 131.9, 129.9, 114.3, 55.6.

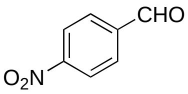

8b

4-Nitrobenzaldehyde (**8b**, 131 mg, 87%), CAS Number 555-16-8. The title compound was prepared according to the general procedure A. ^{1}**H NMR** (600 MHz, $CDCl_3$) δ (ppm): 10.18 (s, 1H), 8.41 (d, *J* = 8.7 Hz, 2H), 8.10 (d, *J* = 8.7 Hz, 2H). ^{13}**C NMR** (151 MHz, $CDCl_3$) δ (ppm): 190.3, 151.2, 140.1, 130.5, 124.3.

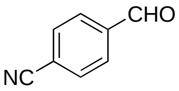

9b

4-Formylbenzonitrile (**9b**, 117 mg, 89%), CAS Number 105-07-7. The title compound was prepared according to the general procedure A. ^{1}**H NMR** (600 MHz, $CDCl_3$) δ (ppm): 10.11 (s, 1H), 8.02 (d, *J* = 8.3 Hz, 2H), 7.87 (d, *J* = 8.3 Hz, 2H). ^{13}**C NMR** (151 MHz, $CDCl_3$) δ (ppm): 190.6, 138.8, 132.9, 129.9, 117.7, 117.6.

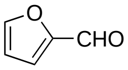

10b

Furan-2-carbaldehyde (**10b**, 69 mg, 24%), CAS Number 98-01-1. The title compound was prepared according to the general procedure A on a 3 mmol scale. ^{1}**H NMR** (600 MHz, $CDCl_3$) δ (ppm): 9.67 (s, 1H), 7.72–7.68 (m, 1H), 7.26 (dd, *J* = 3.6, 0.8 Hz, 1H), 6.61 (dd, *J* = 3.6, 1.7 Hz, 1H). ^{13}**C NMR** (151 MHz, $CDCl_3$) δ (ppm): 177.9, 153.0, 148.1, 120.9, 112.6.

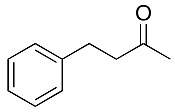

11b

4-Phenylbutan-2-one (**11b**, 136 mg, 92%), CAS Number 2550-26-7. The title compound was prepared according to the general procedure B. **^{1}H NMR** (600 MHz, $CDCl_3$) δ (ppm): 7.30 (q, *J* = 7.2, 6.7 Hz, 2H), 7.22 (t, *J* = 8.2 Hz, 3H), 2.93 (t, *J* = 7.7 Hz, 2H), 2.79 (t, *J* = 7.7 Hz, 2H), 2.17 (s, 3H). **^{13}C NMR** (151 MHz, $CDCl_3$) δ (ppm): 208.0, 141.0, 128.5, 128.3, 126.1, 45.2, 30.1, 29.8.

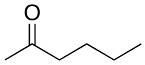

12b

Hexan-2-one (**12b**, 80 mg, 80%), CAS Number 591-78-6. The title compound was prepared according to the general procedure B. **^{1}H NMR** (600 MHz, $CDCl_3$) δ (ppm): 2.43 (t, *J* = 7.5 Hz, 2H), 2.14 (s, 3H), 1.57 (p, *J* = 7.5 Hz, 2H), 1.33 (dt, *J* = 14.9, 7.4 Hz, 2H), 0.92 (t, *J* = 7.4 Hz, 3H). **^{13}C NMR** (151 MHz, $CDCl_3$) δ (ppm): 209.3, 43.5, 29.8, 26.0, 22.3, 13.8.

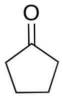

13b

Cyclopentanone (**13b**, 120 mg, 71%), CAS Number 120-92-3. The title compound was prepared according to the general procedure B on a two mmol scale. **^{1}H NMR (**600 MHz, $CDCl_3$) δ (ppm): 2.20–2.13 (m, 4H), 1.99–1.92 (m, 4H). **^{13}C NMR** (151 MHz, $CDCl_3$) δ (ppm): 220.6, 38.3, 23.2.

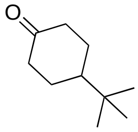

14b

4-(*tert*-Butyl)cyclohexan-1-one (**14b**, 134 mg, 87%), CAS Number 98-53-3. The title compound was prepared according to the general procedure B. **^{1}H NMR** (600 MHz, $CDCl_3$) δ (ppm): 2.42-2.40 (m, 2H), 2.15-2.09 (m, 2H), 2.13–2.06 (m, 2H), 1.49-1.45 (m, 3H), 0.93 (s, 9H). **^{13}C NMR** (151 MHz, $CDCl_3$) δ (ppm): 212.6, 46.7, 41.3, 32.5, 27.6 (2xC).

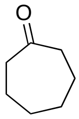

15b

Cycloheptanone (**15b**, 95 mg, 85%), CAS Number 502-42-1. The title compound was prepared according to the general procedure B. **^{1}H NMR** (600 MHz, $CDCl_3$) δ (ppm): 2.53–2.47 (m, 4H), 1.76–1.65 (m, 8H). **^{13}C NMR** (151 MHz, $CDCl_3$) δ (ppm): 215.4, 43.9, 30.4, 24.3.

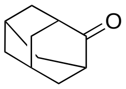

16b

Adamantan-2-one (**16b**, 125 mg, 83%), CAS Number 700-58-3. The title compound was prepared according to the general procedure B by adding Azado catalyst (3.4 mg, 2 mol %). After 60 min the reaction was almost complete by GC-MS analyses. **^{1}H**

NMR (600 MHz, $CDCl_3$) δ (ppm): 2.55 (bs, 2H), 2.16–2.05 (m, 4H), 2.03-1.97 (m, 6H), 1.95-1.92 (bs, 2H). **^{13}C NMR** (151 MHz, $CDCl_3$) δ (ppm): 218.4, 47.0, 39.3, 36.3, 27.5.

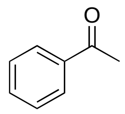

17b

Acetophenone (**17b**, 109 mg, 91%), CAS Number 98-86-2. The title compound was prepared according to the general procedure B without adding AZADO catalyst. After 15 min the reaction was almost complete by GC-MS analyses. **^{1}H NMR** (600 MHz, $CDCl_3$) δ (ppm): 7.98 (dt, J = 8.5, 1.1 Hz, 2H), 7.59 (tq, J = 7.9, 1.3 Hz, 1H), 7.49 (tt, J = 7.4, 1.5 Hz, 2H), 2.63 (s, 3H). **^{13}C NMR** (151 MHz, $CDCl_3$) δ (ppm): 198.2, 137.2, 133.1, 128.6, 128.3, 26.7.

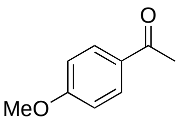

18b

1-(4-Methoxyphenyl)ethan-1-one (**18b**, 134 mg, 89%), CAS Number 100-06-1 The title compound was prepared according to the general procedure B without adding AZADO catalyst. After 15 minutes, the reaction was almost complete by GC-MS analyses. **^{1}H NMR** (600 MHz, $CDCl_3$) δ (ppm): 7.94 (d, J = 8.8 Hz, 2H), 6.94 (d, J = 8.8 Hz, 2H), 3.87 (s, 3H), 2.56 (s, 3H). **^{13}C NMR** (151 MHz, $CDCl_3$) δ (ppm): 196.7, 163.5, 130.6, 130.4, 113.7, 55.4, 26.3.

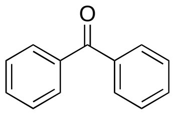

19b

Benzophenone (**19b**, 170 mg, 93%), CAS Number 119-61-9 . The title compound was prepared according to the general procedure B without adding AZADO catalyst. After 15 minutes, the reaction was almost complete by GC-MS analyses. **^{1}H NMR** (600 MHz, $CDCl_3$) δ (ppm): 7.83 (dd, J = 8.2, 1.4 Hz, 4H), 7.66–7.58 (td, J= 7.7 Hz, 1.4 Hz, 2H), 7.51 (t, J = 7.7 Hz, 4H). **^{13}C NMR** (151 MHz, $CDCl_3$) δ (ppm): 196.7, 137.7, 132.4, 130.1, 128.3.

6. ^{1}H and ^{13}C NMR Spectra for carbonyl compounds **1b-19b**.

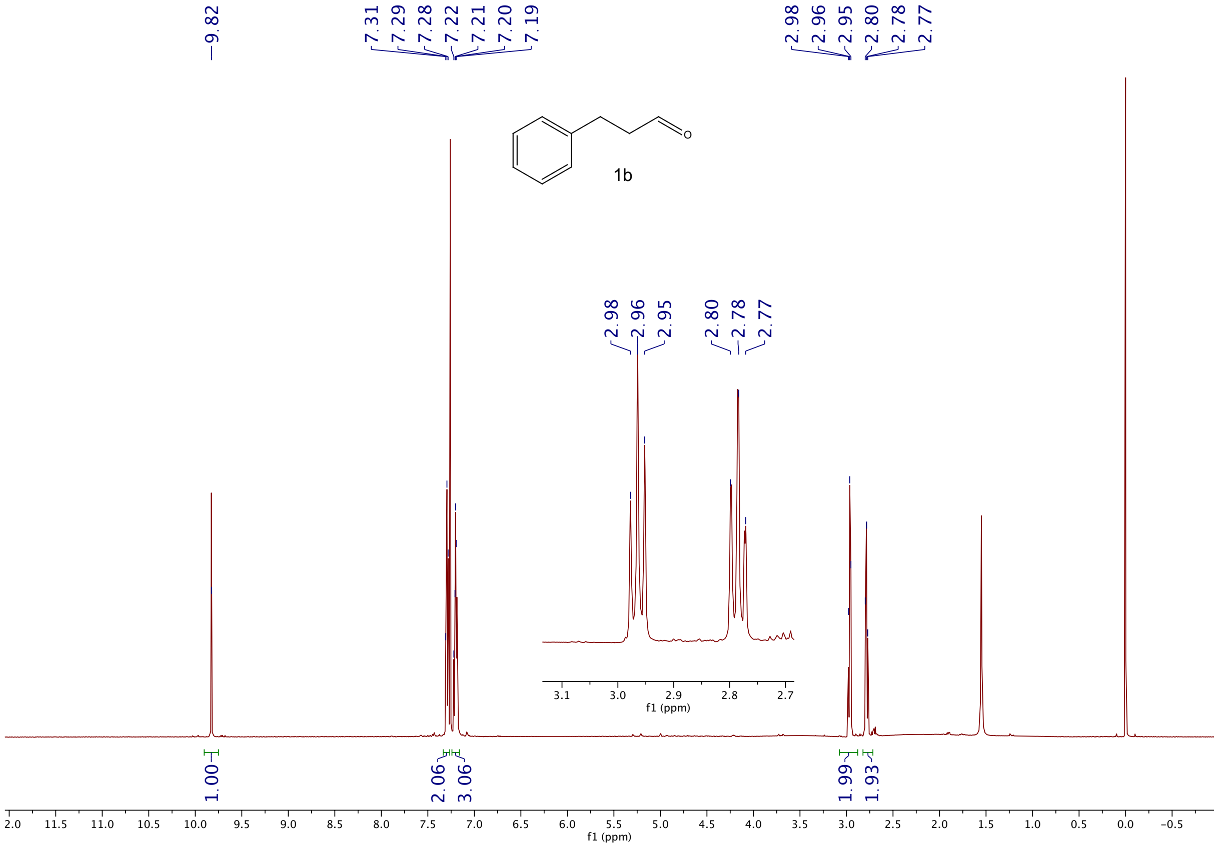

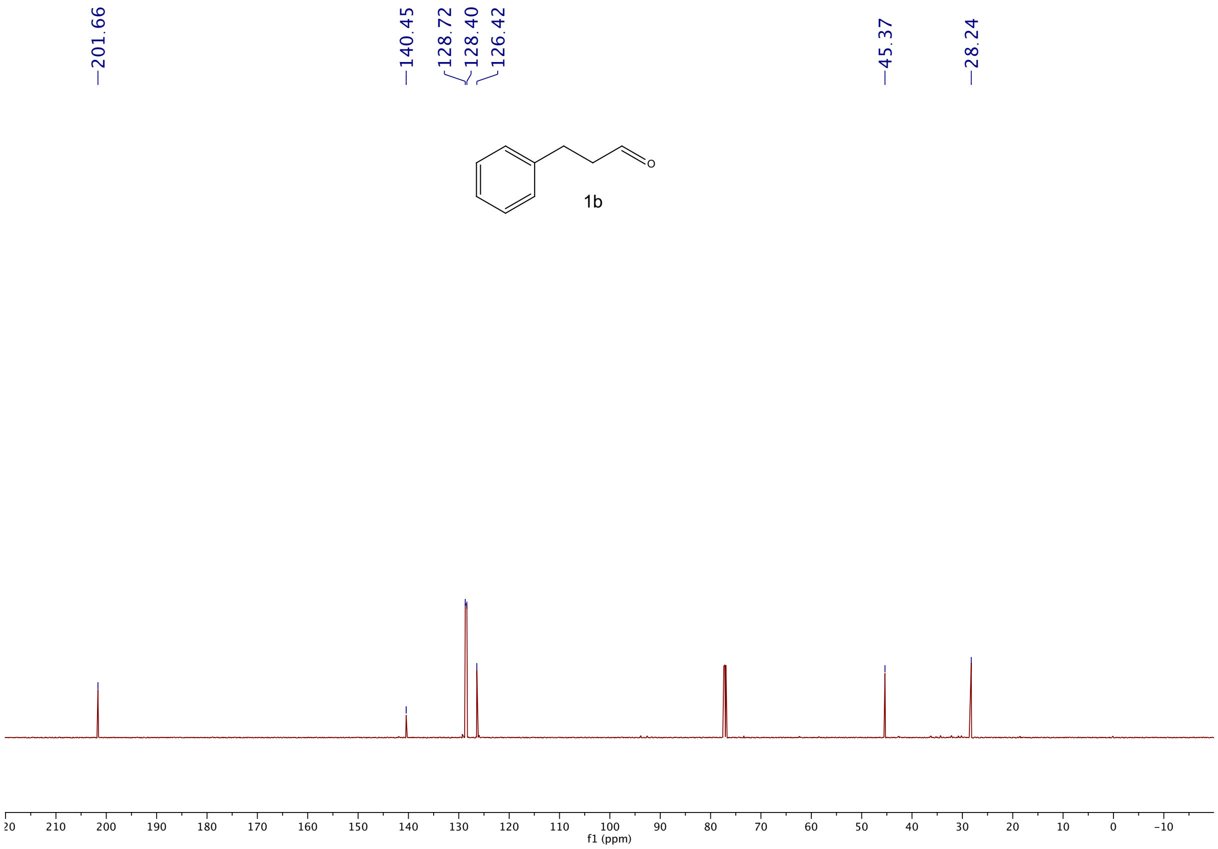

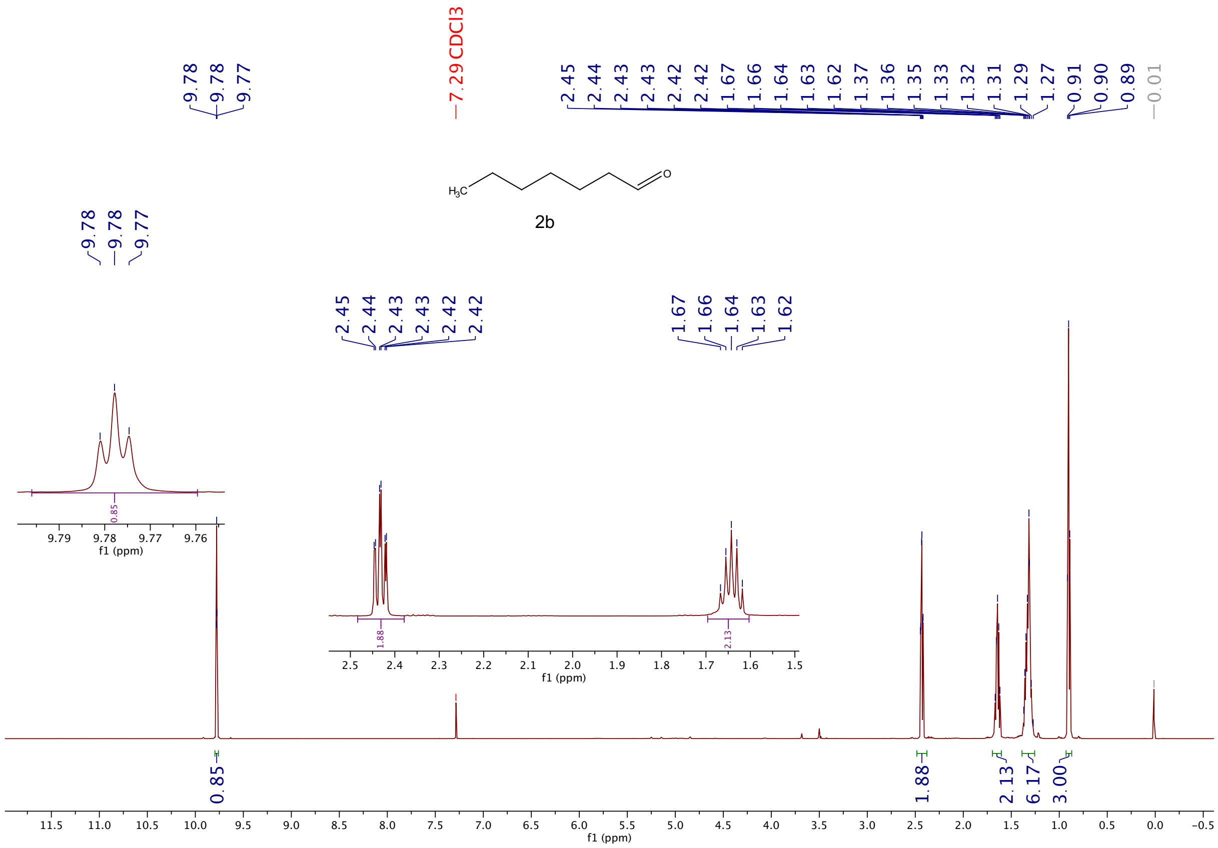
9.78
9.78
9.77
7.29 CDCl3
2.45
2.44
2.43
2.43
2.42
2.42
1.67
1.66
1.64
1.63
1.62
1.37
1.36
1.35
1.33
1.32
1.31
1.29
1.27
0.91
0.90
0.89
0.01
H_3C
O
2b
0.85
1.88
2.13
6.17
3.00
f1 (ppm)

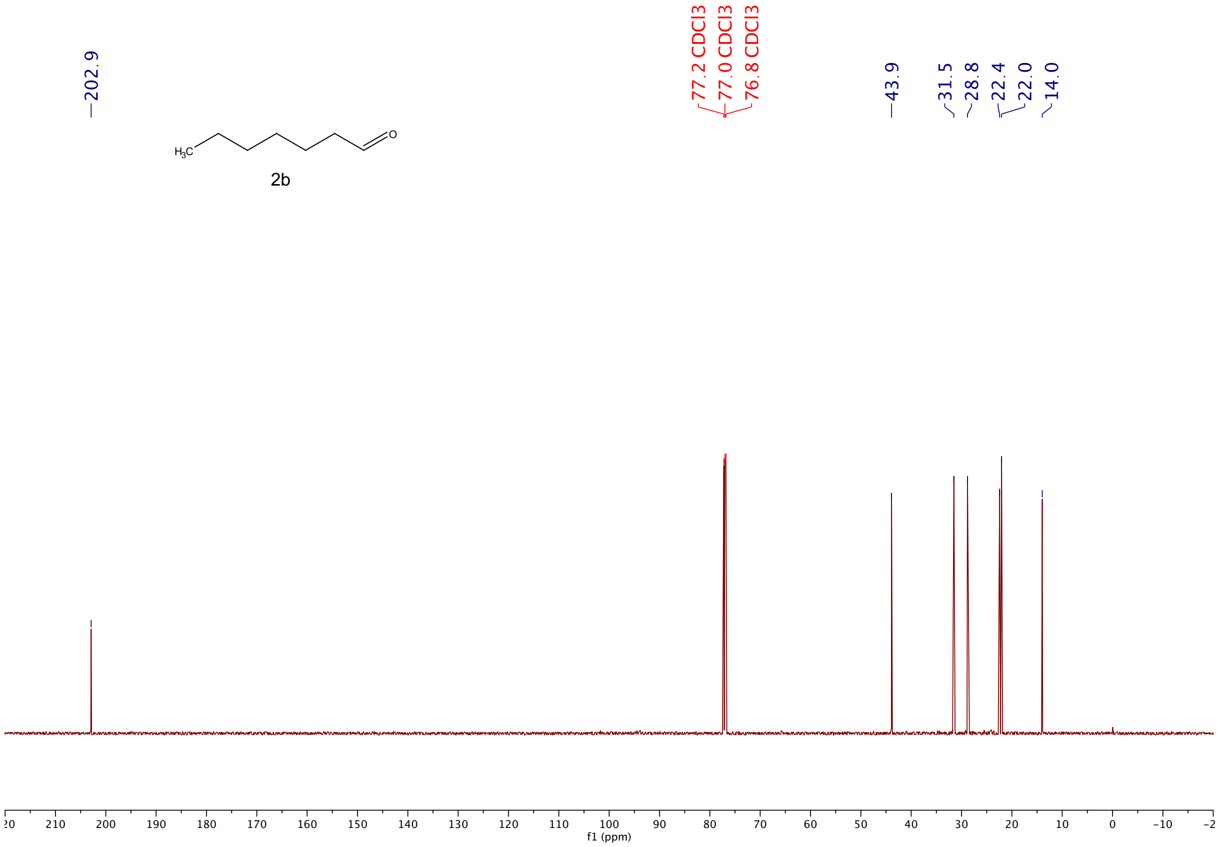
202.9
77.2 CDCl3
77.0 CDCl3
76.8 CDCl3
43.9
31.5
28.8
22.4
22.0
14.0
H_3C
O
2b
f1 (ppm)

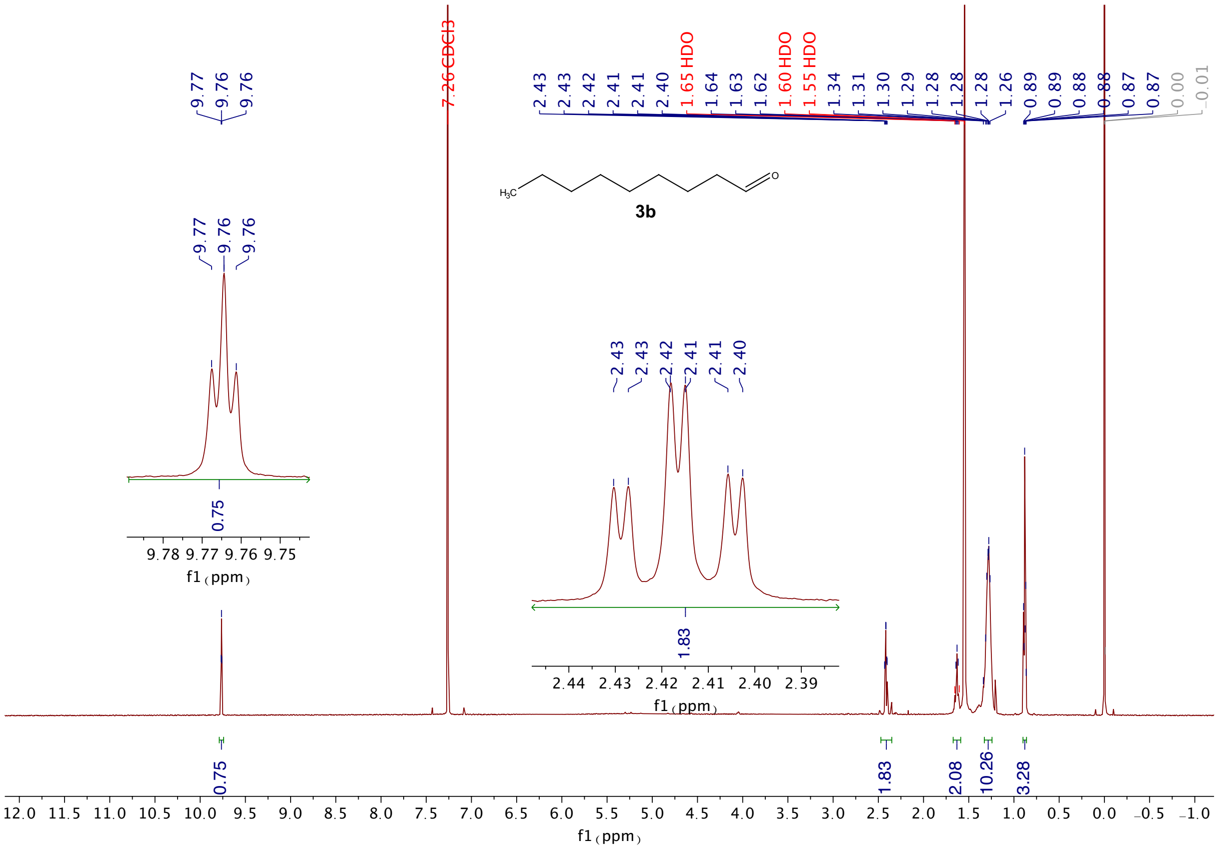

9.77
9.76
9.76
7.26 CDCl3
2.43
2.43
2.42
2.41
2.41
2.40
1.65 HDO
1.64
1.63
1.62
1.60 HDO
1.55 HDO
1.34
1.31
1.30
1.29
1.28
1.28
1.28
1.26
0.89
0.89
0.88
0.88
0.87
0.87
0.00
-0.01
3b
0.75
1.83
2.08
10.26
3.28
f1 (ppm)

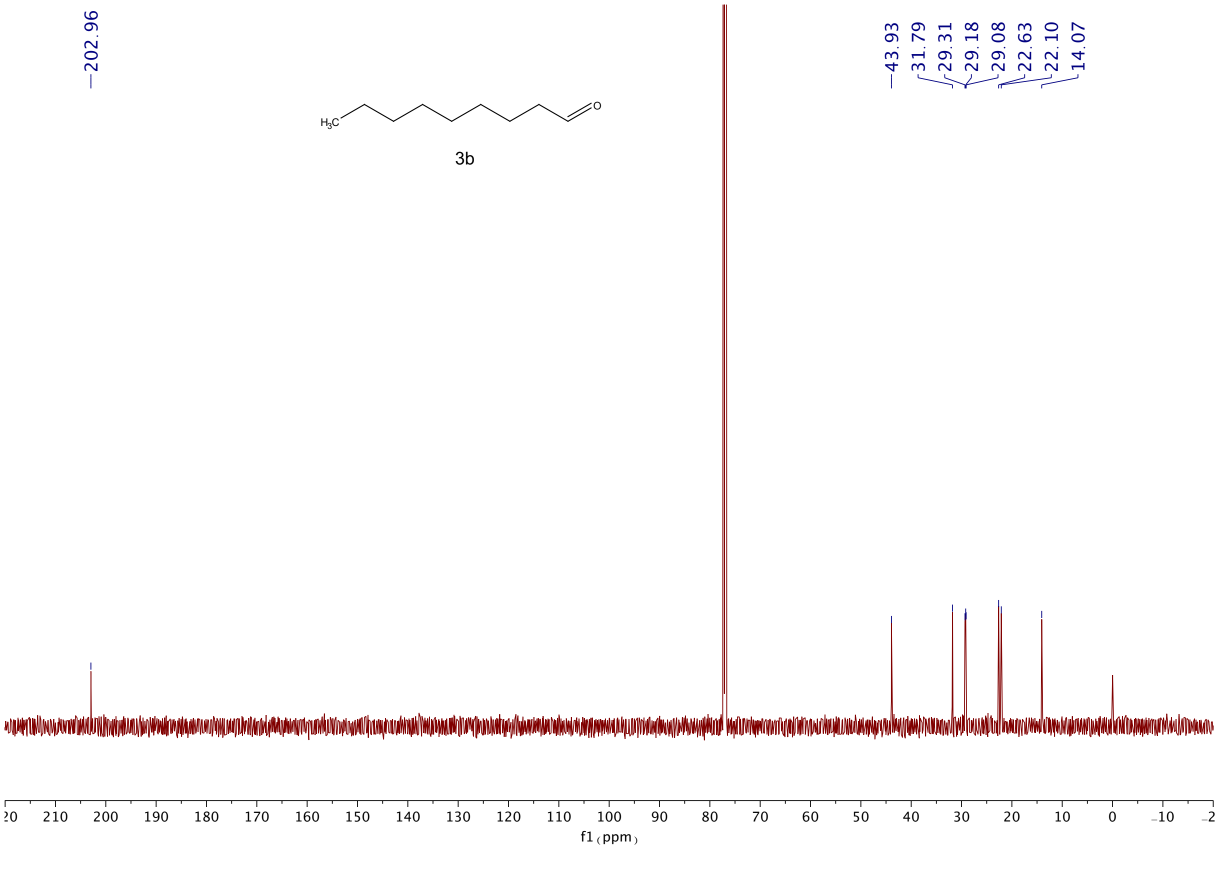

202.96
43.93
31.79
29.31
29.18
29.08
22.63
22.10
14.07
3b
f1 (ppm)

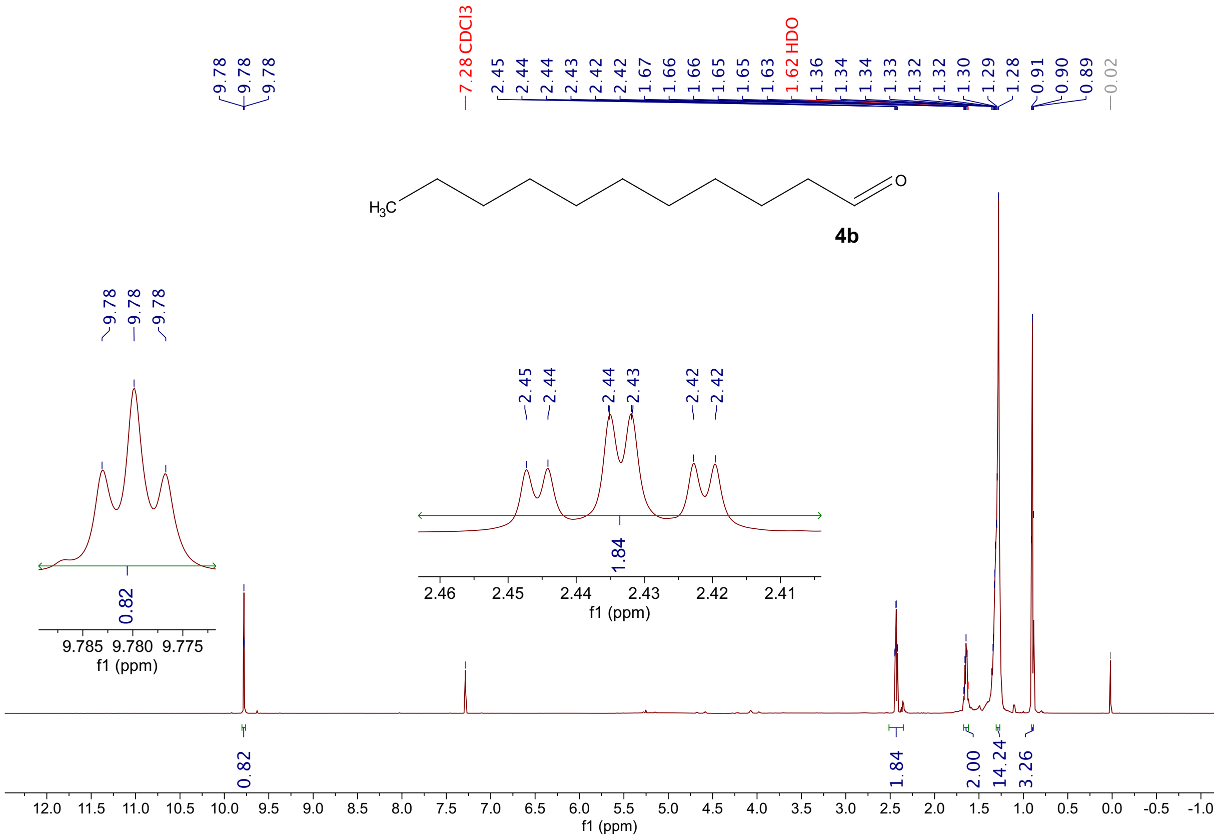
9.78
9.78
9.78
7.28 CDCl3
2.45
2.44
2.44
2.43
2.42
2.42
1.67
1.66
1.66
1.65
1.65
1.63
1.62 HDO
1.36
1.34
1.34
1.33
1.32
1.32
1.30
1.29
1.28
0.91
0.90
0.89
0.02
H3C
O
4b
0.82
9.785 9.780 9.775
f1 (ppm)
1.84
2.46 2.45 2.44 2.43 2.42 2.41
0.82
1.84
2.00
14.24
3.26
12.0 11.5 11.0 10.5 10.0 9.5 9.0 8.5 8.0 7.5 7.0 6.5 6.0 5.5 5.0 4.5 4.0 3.5 3.0 2.5 2.0 1.5 1.0 0.5 0.0 -0.5 -1.0

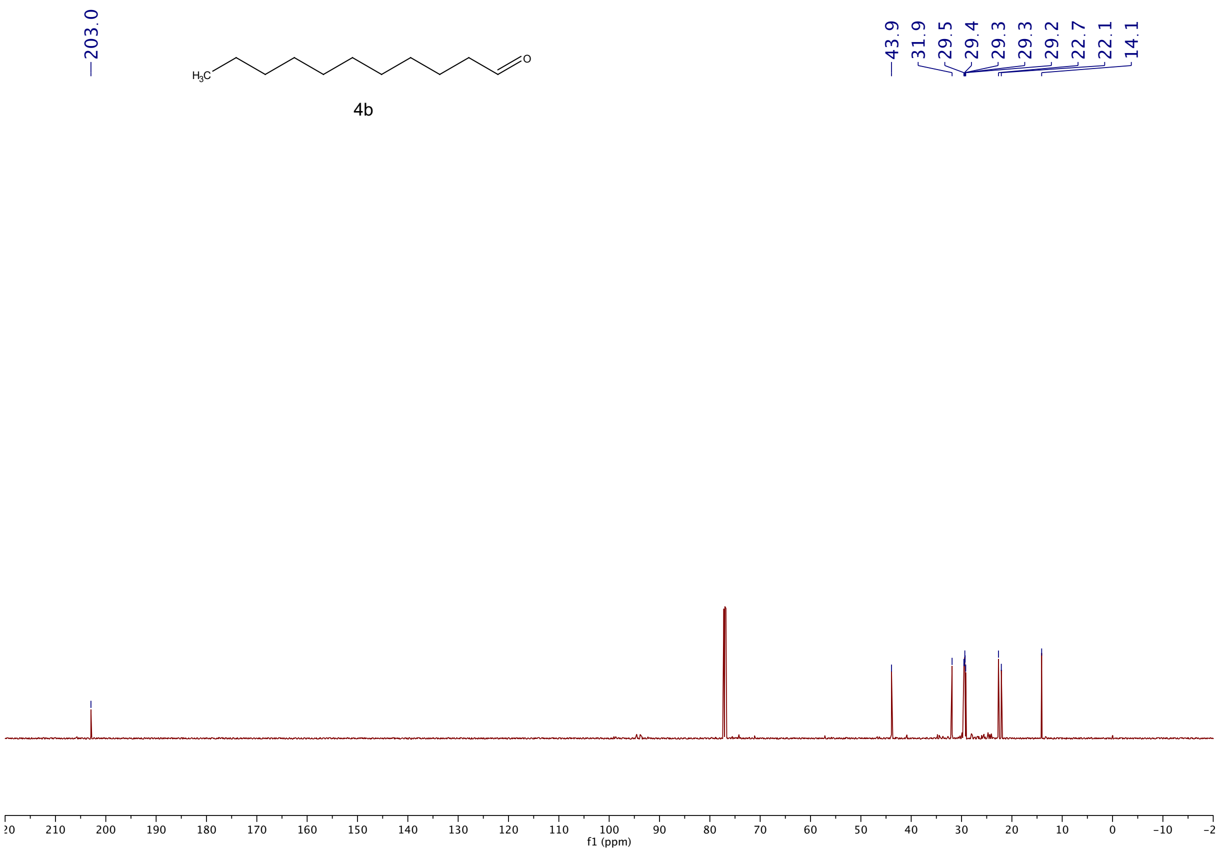
203.0
H3C
O
4b
43.9
31.9
29.5
29.4
29.3
29.3
29.2
22.7
22.1
14.1
210 200 190 180 170 160 150 140 130 120 110 100 90 80 70 60 50 40 30 20 10 0 −10
f1 (ppm)

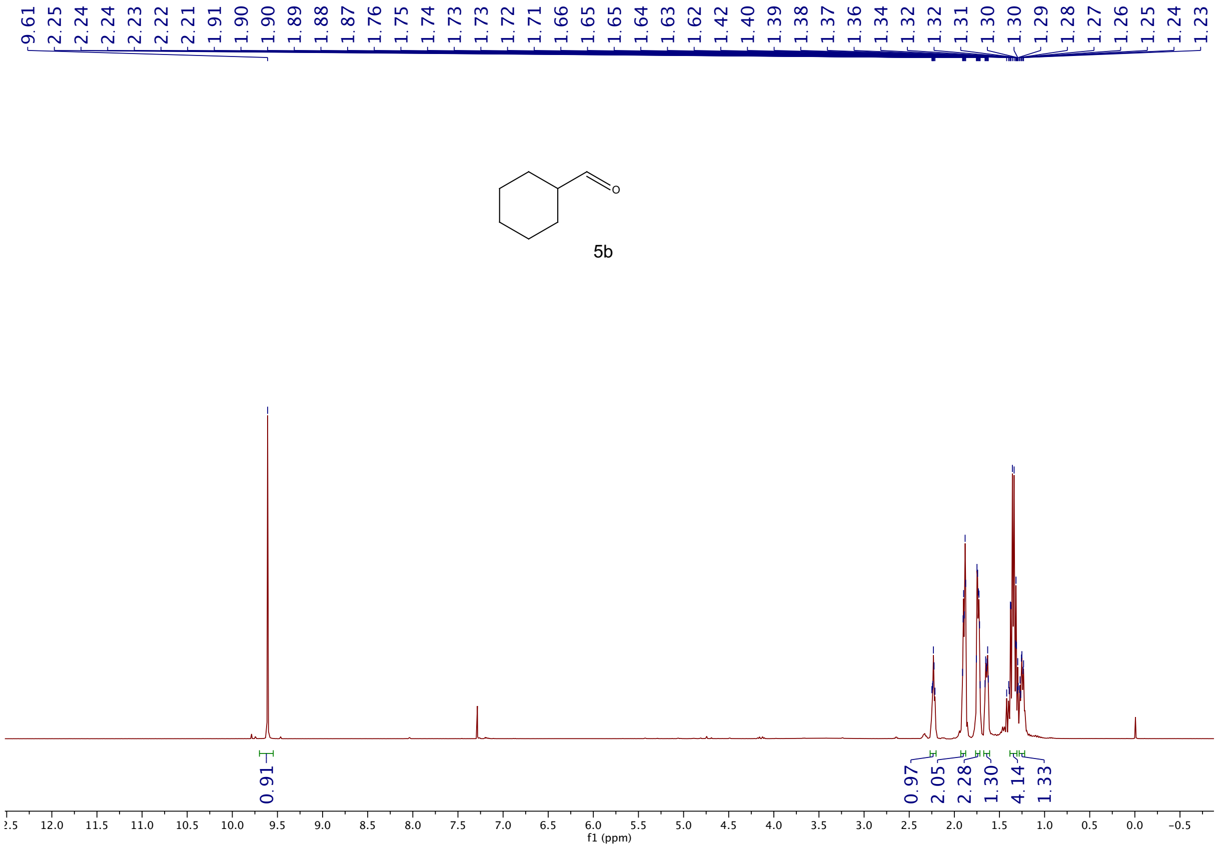
9.61 2.25 2.24 2.24 2.23 2.22 2.21 1.91 1.90 1.90 1.89 1.88 1.87 1.76 1.75 1.74 1.73 1.73 1.72 1.71 1.66 1.65 1.65 1.64 1.63 1.62 1.42 1.40 1.39 1.38 1.37 1.36 1.34 1.32 1.32 1.31 1.30 1.30 1.29 1.28 1.27 1.26 1.25 1.24 1.23
5b
0.91
0.97
2.05
2.28
1.30
4.14
1.33
f1 (ppm)

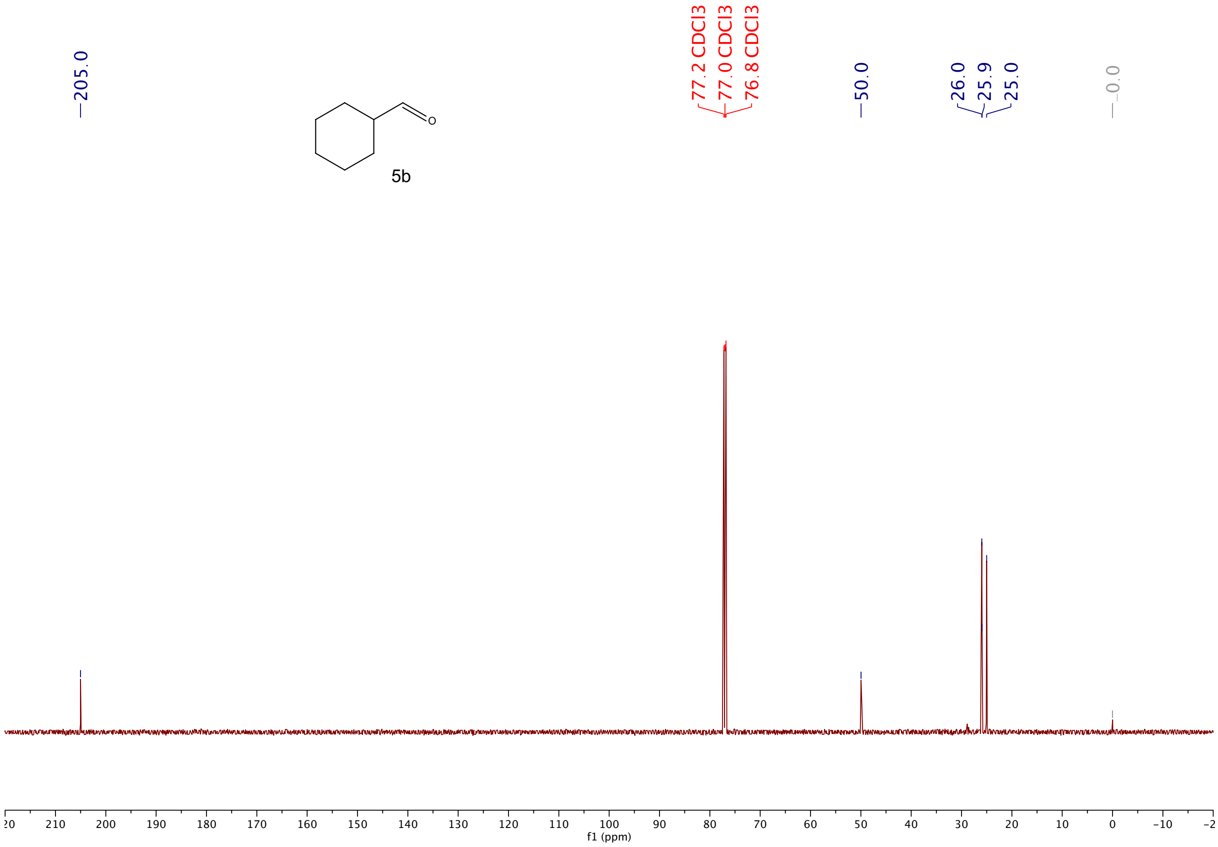
205.0
77.2 CDCl3
77.0 CDCl3
76.8 CDCl3
50.0
26.0
25.9
25.0
0.0
5b
f1 (ppm)

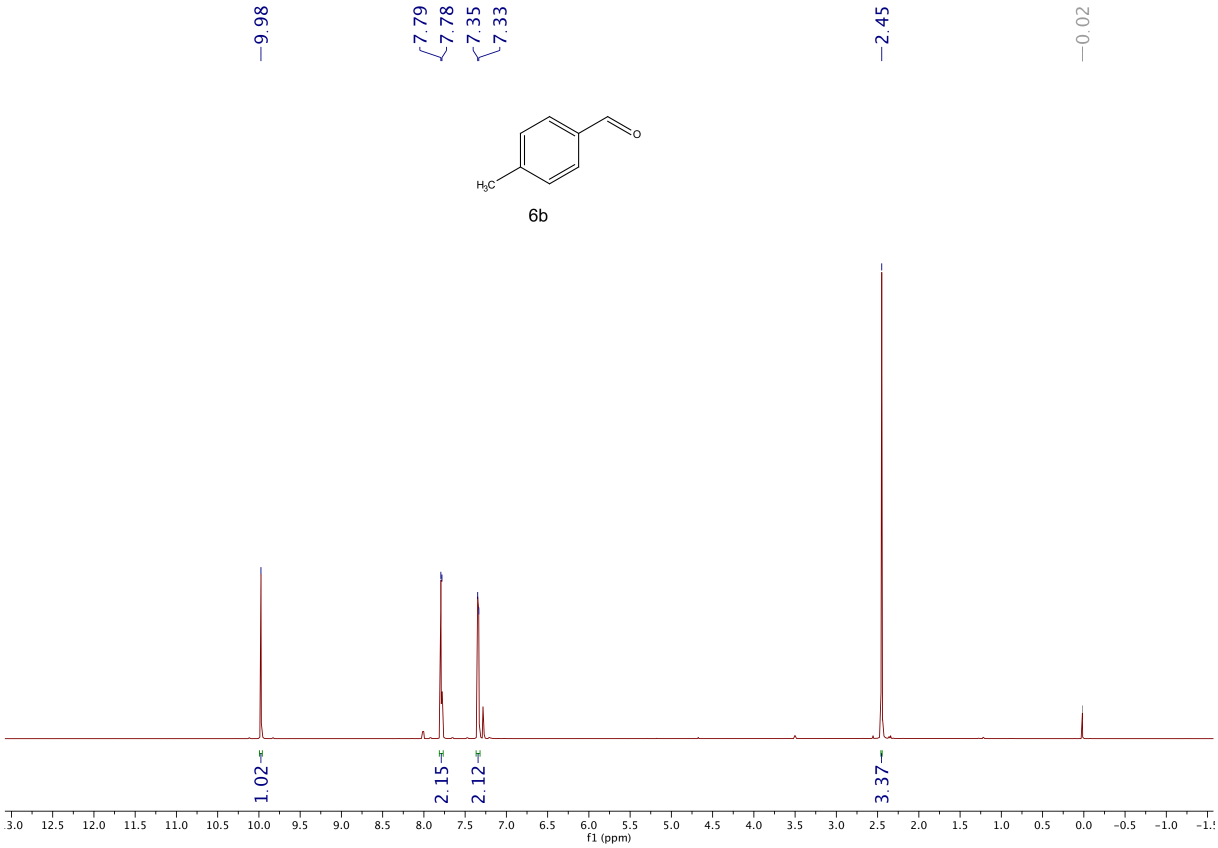
9.98
7.79
7.78
7.35
7.33
2.45
0.02
H3C
O
6b
1.02
2.15
2.12
3.37
f1 (ppm)

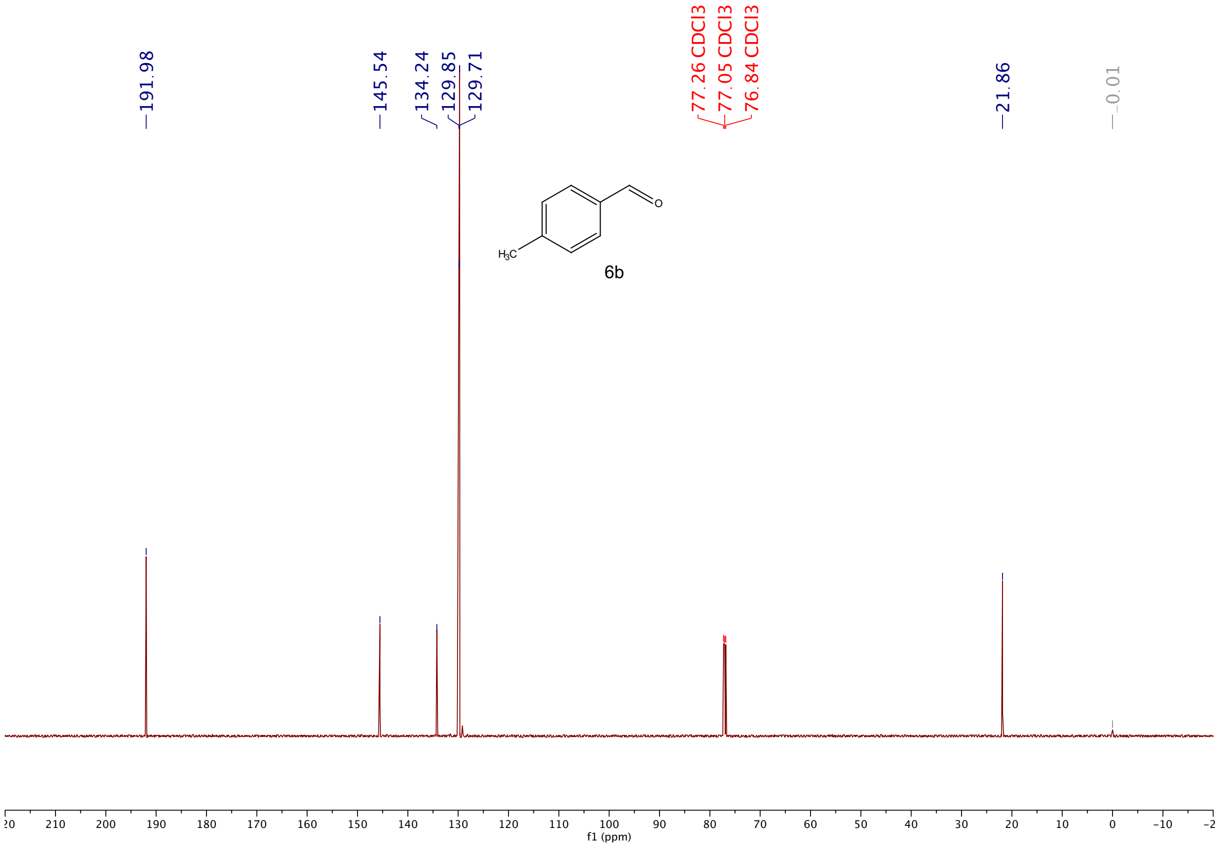
191.98
145.54
134.24
129.85
129.71
77.26 CDCl3
77.05 CDCl3
76.84 CDCl3
21.86
0.01
H3C
O
6b
f1 (ppm)

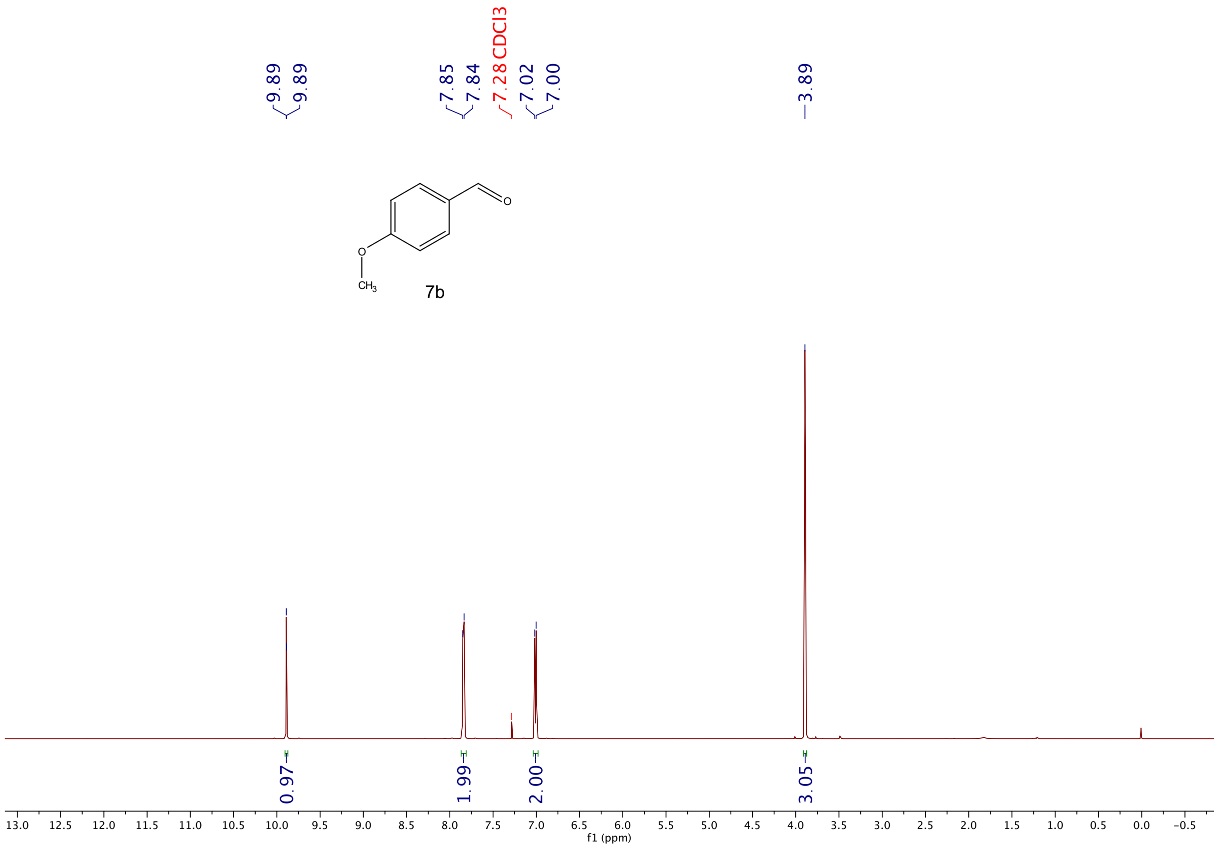

9.89
9.89
7.85
7.84
7.28 CDCl3
7.02
7.00
3.89
O
O
CH3
7b
0.97
1.99
2.00
3.05
13.0 12.5 12.0 11.5 11.0 10.5 10.0 9.5 9.0 8.5 8.0 7.5 7.0 6.5 6.0 5.5 5.0 4.5 4.0 3.5 3.0 2.5 2.0 1.5 1.0 0.5 0.0 -0.5
f1 (ppm)

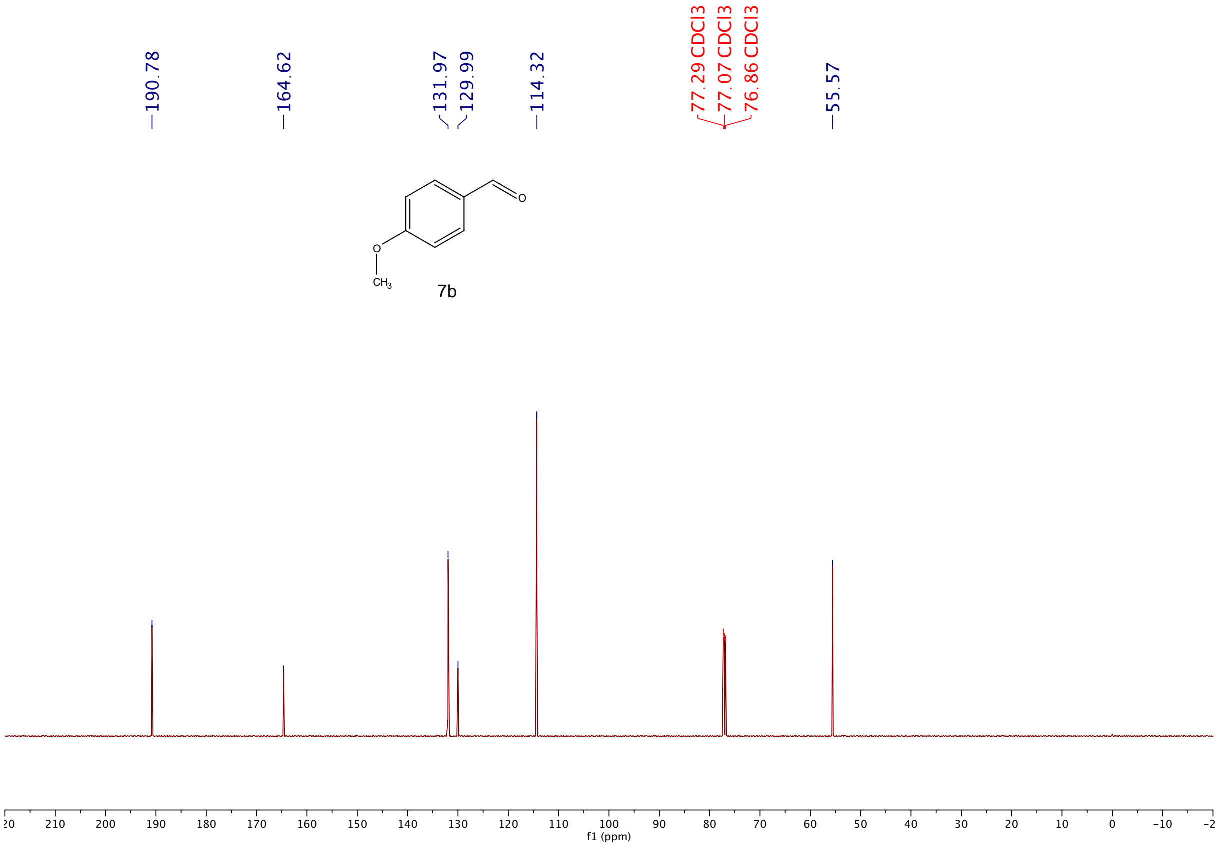

190.78
164.62
131.97
129.99
114.32
77.29 CDCl3
77.07 CDCl3
76.86 CDCl3
55.57
O
O
CH3
7b
210 200 190 180 170 160 150 140 130 120 110 100 90 80 70 60 50 40 30 20 10 0 -10
f1 (ppm)

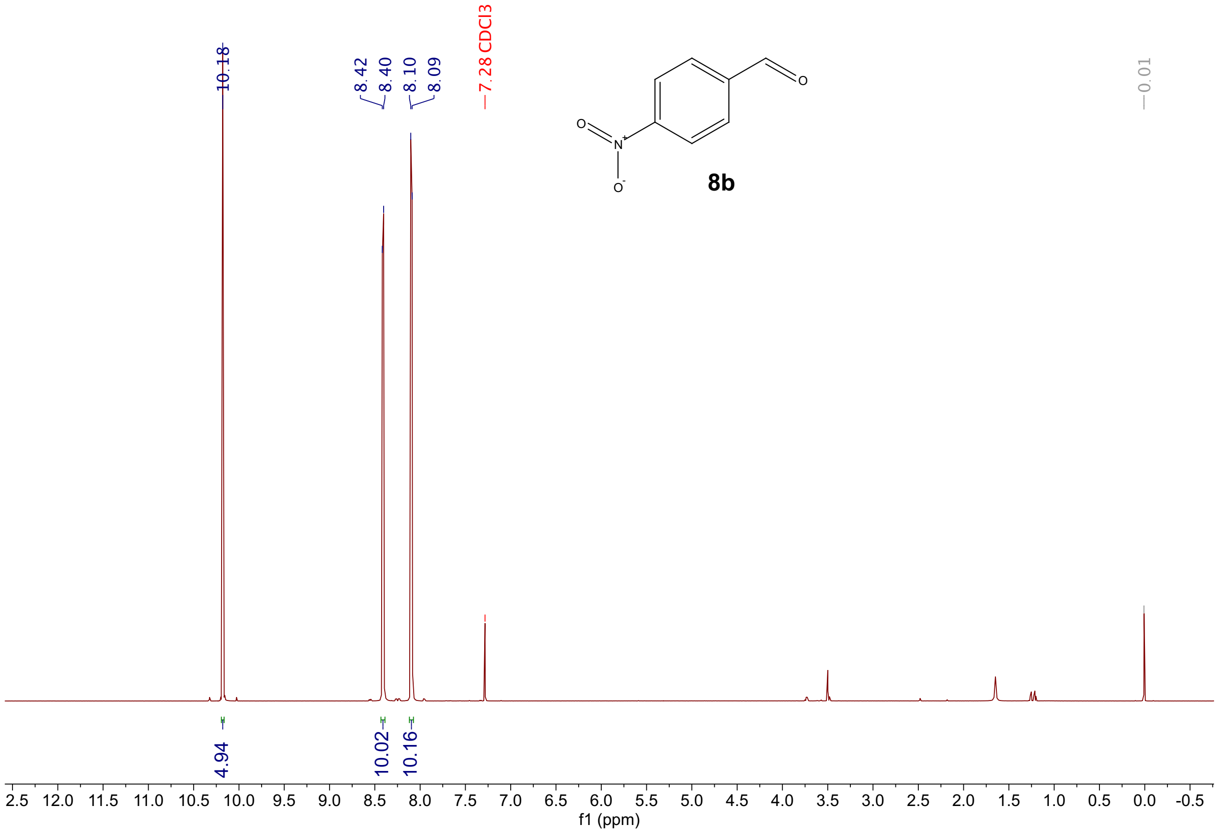
10.18
8.42
8.40
8.10
8.09
7.28 CDCl3
-0.01
O
N+
O-
O
8b
4.94
10.02
10.16
f1 (ppm)

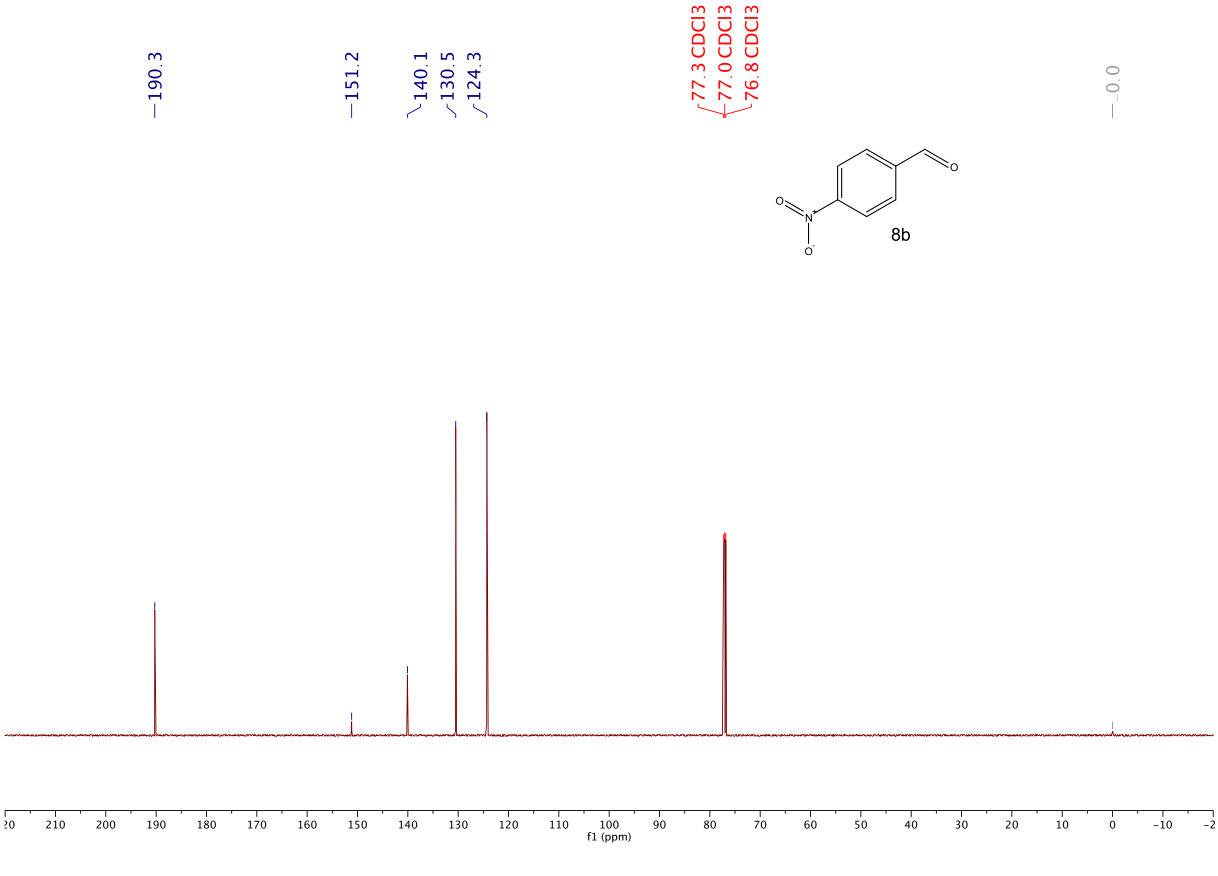
190.3
151.2
140.1
130.5
124.3
77.3 CDCl3
77.0 CDCl3
76.8 CDCl3
0.0
O
N+
O-
O
8b
f1 (ppm)

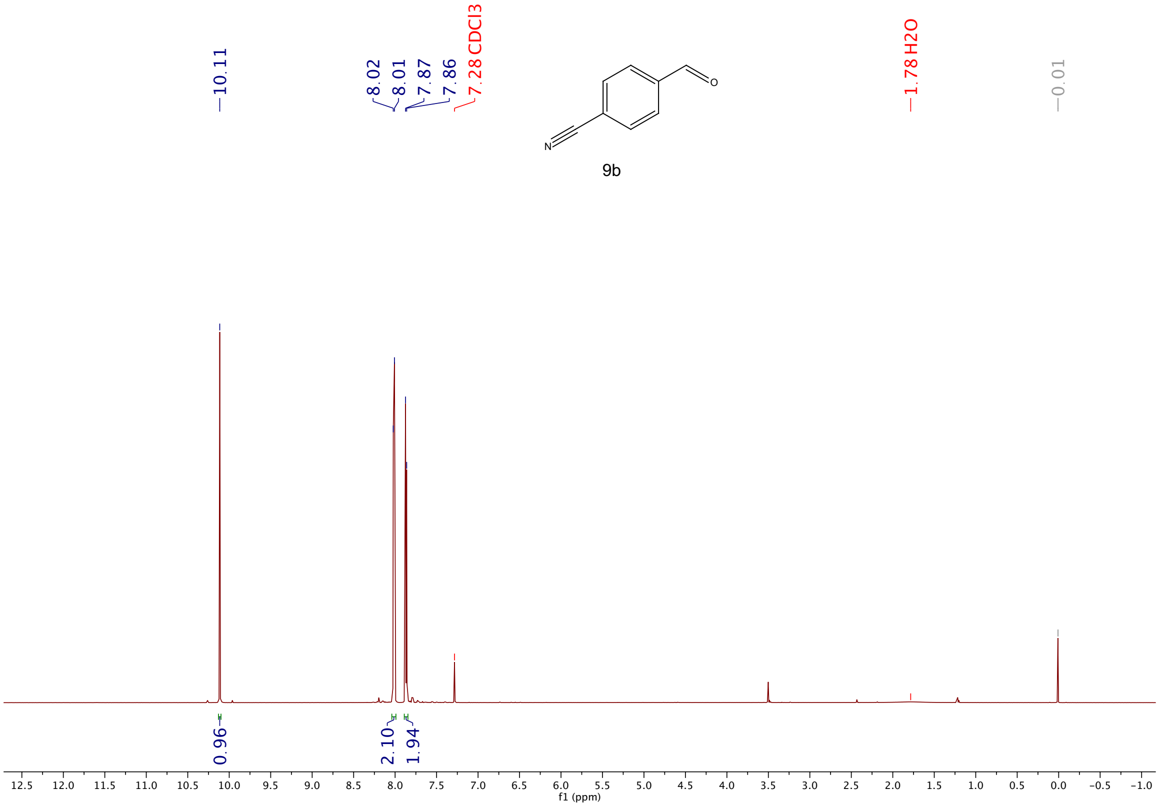
10.11
8.02
8.01
7.87
7.86
7.28 CDCl3
1.78 H2O
0.01
N
O
9b
0.96
2.10
1.94
f1 (ppm)

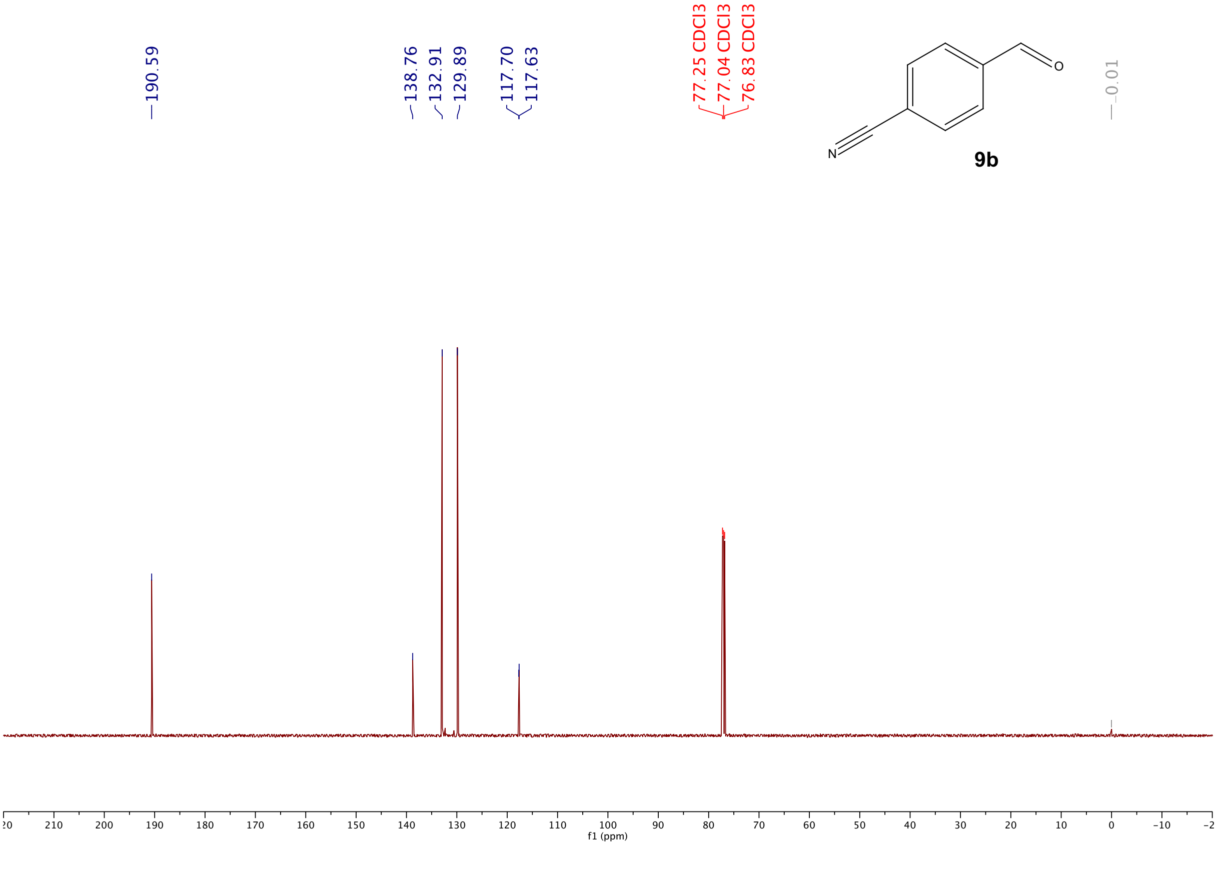
190.59
138.76
132.91
129.89
117.70
117.63
77.25 CDCl3
77.04 CDCl3
76.83 CDCl3
0.01
N
O
9b
f1 (ppm)

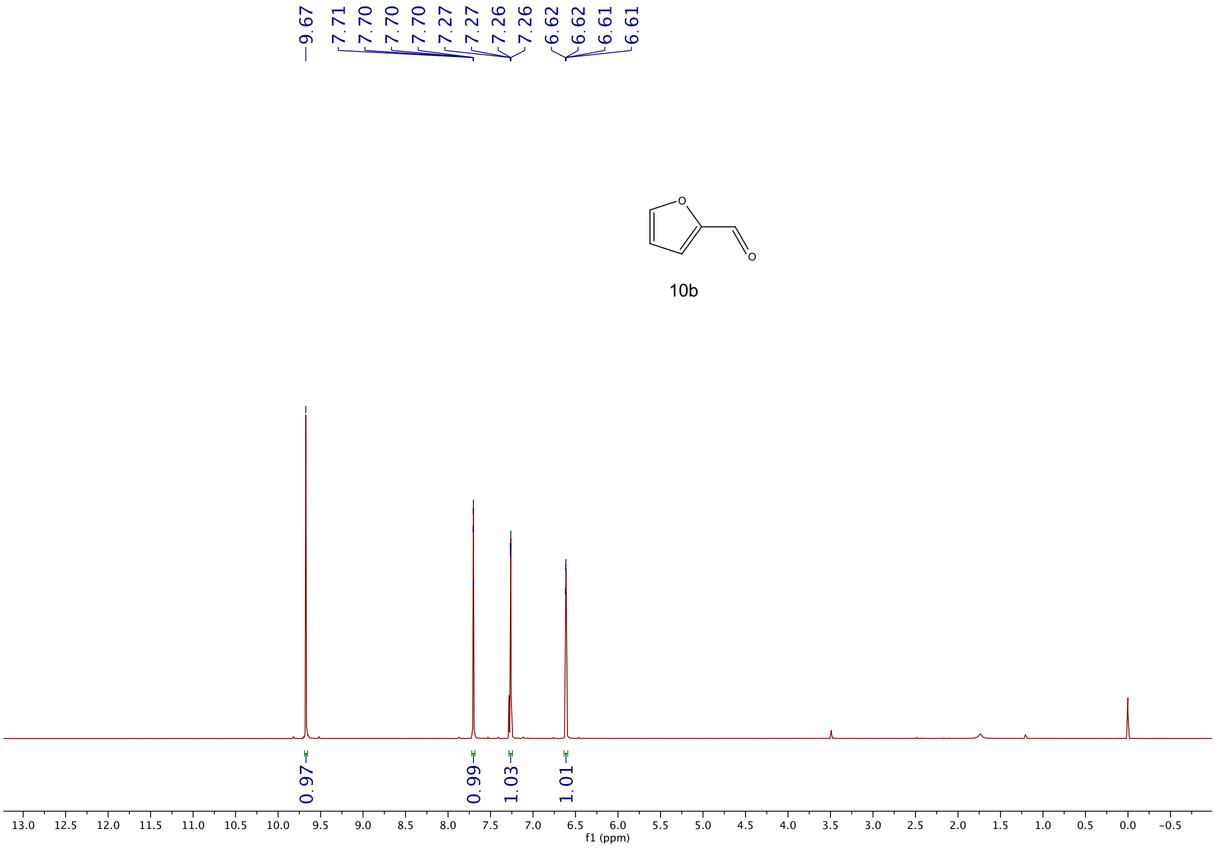
9.67
7.71
7.70
7.70
7.70
7.27
7.27
7.26
7.26
6.62
6.62
6.61
6.61
0.97
0.99
1.03
1.01
f1 (ppm)

10b

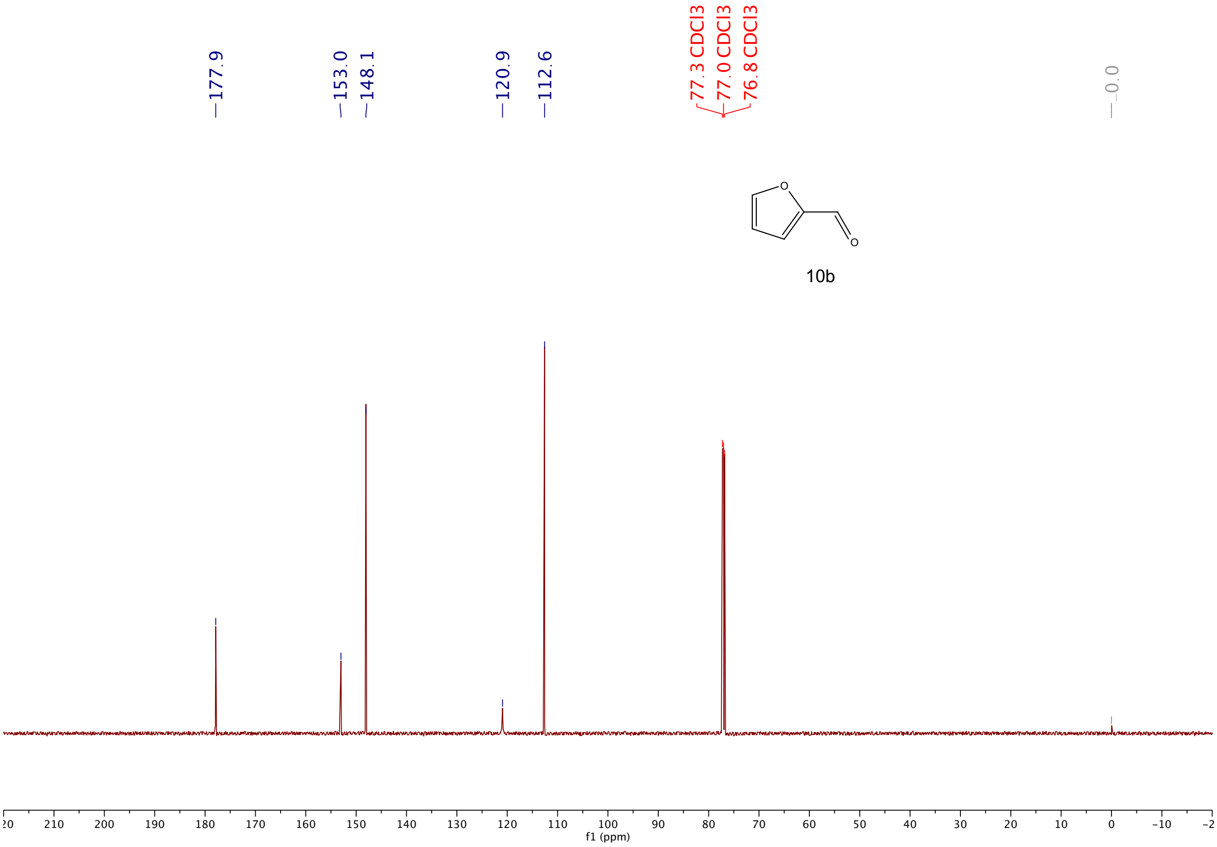
177.9
153.0
148.1
120.9
112.6
77.3 CDCl3
77.0 CDCl3
76.8 CDCl3
0.0
f1 (ppm)

10b

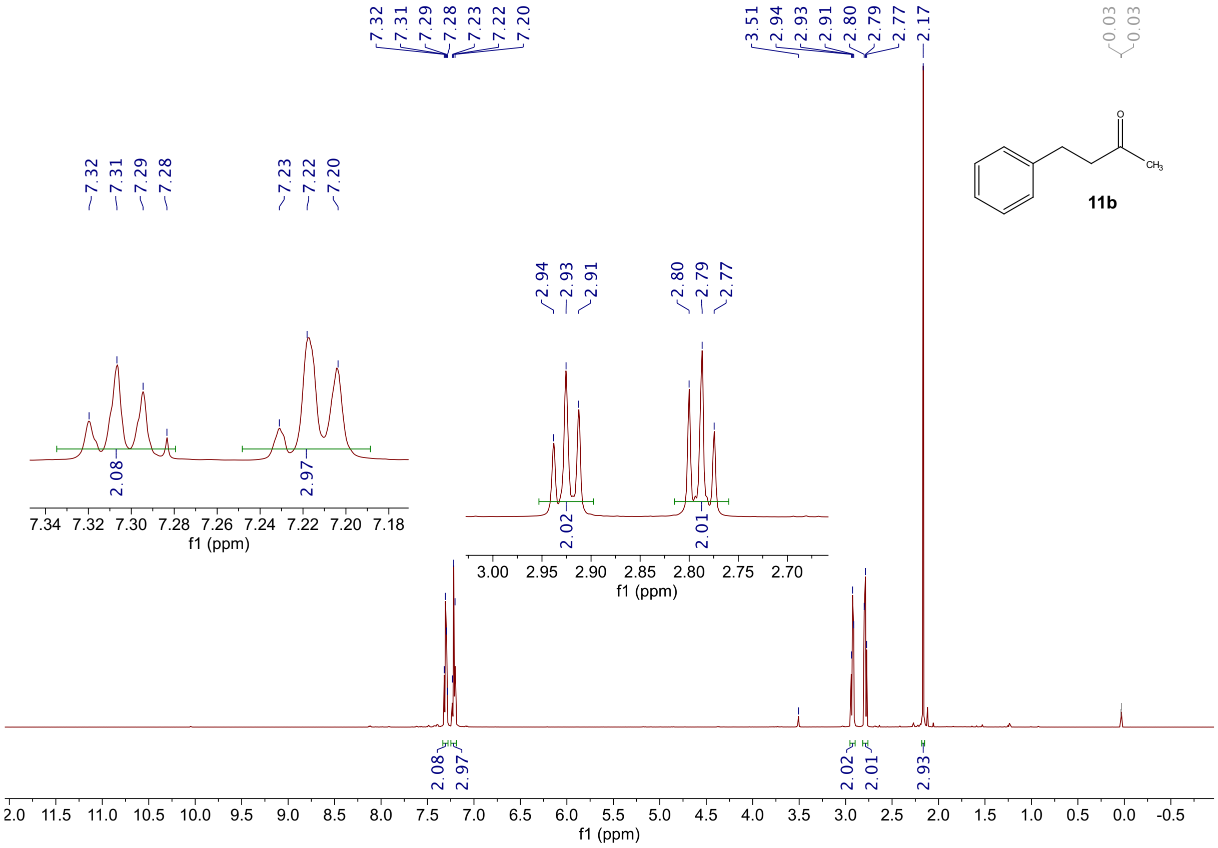
7.32
7.31
7.29
7.28
7.23
7.22
7.20
3.51
2.94
2.93
2.91
2.80
2.79
2.77
2.17
0.03
0.03
O
CH3
11b
2.08
2.97
2.02
2.01
2.93
f1 (ppm)

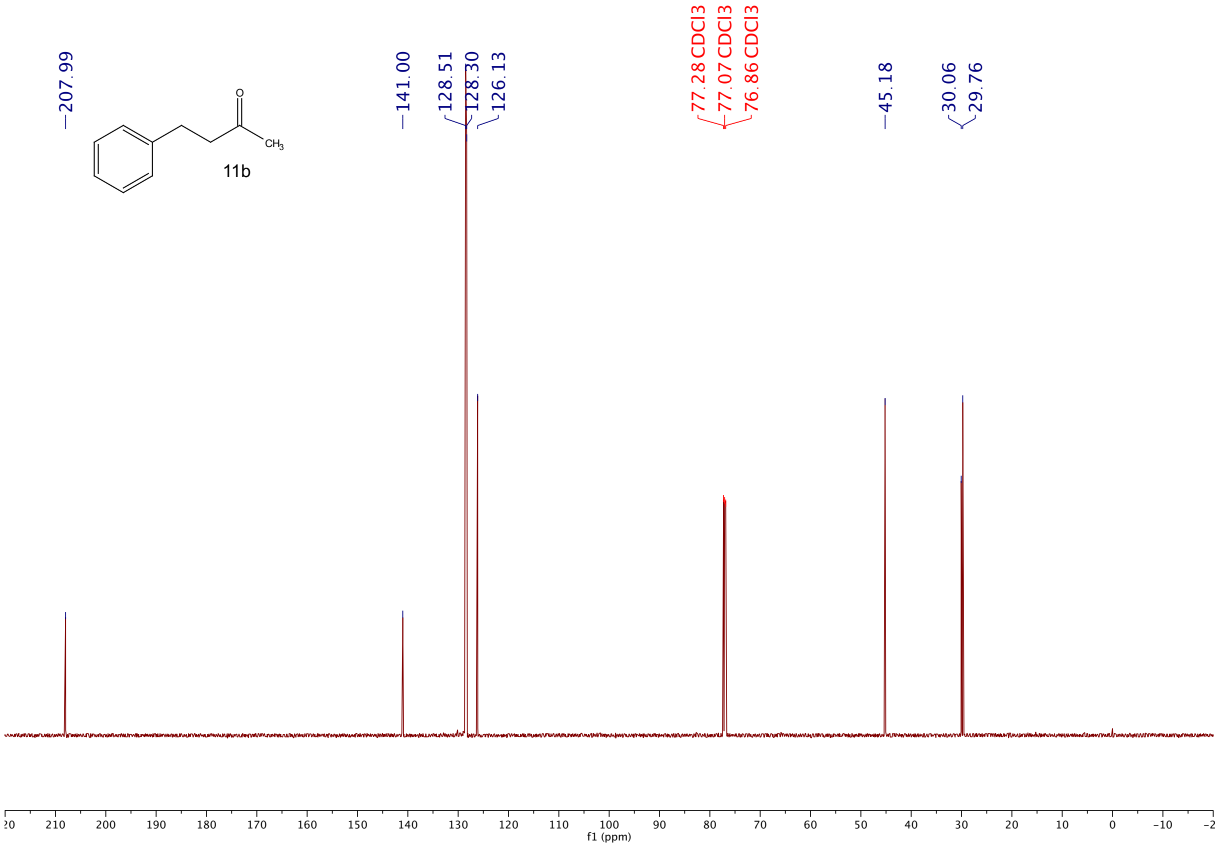
207.99
141.00
128.51
128.30
126.13
77.28 CDCl3
77.07 CDCl3
76.86 CDCl3
45.18
30.06
29.76
O
CH3
11b
f1 (ppm)

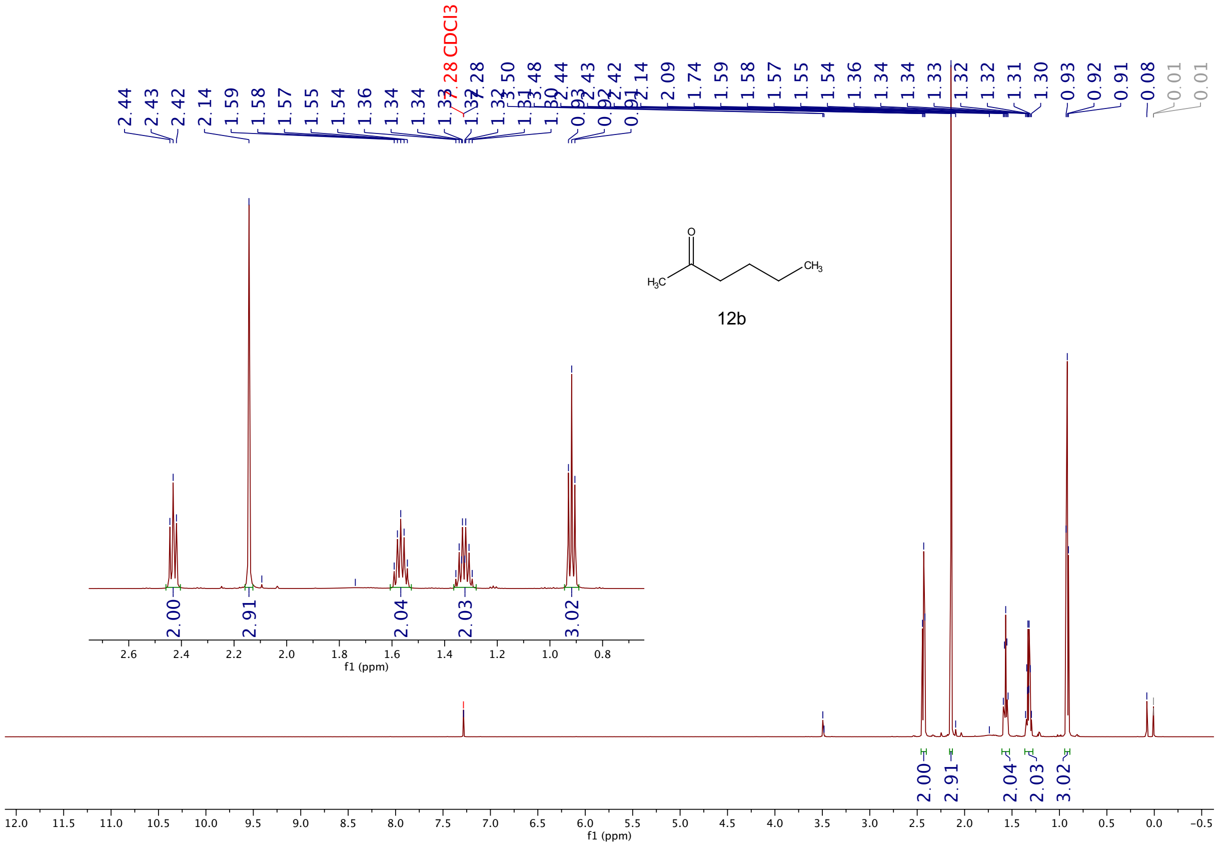
2.44 2.43 2.42 2.14 1.59 1.58 1.57 1.55 1.54 1.36 1.34 1.34 1.33 7.28 CDCl3 1.32 1.32 1.31 1.30 0.93 0.92 0.91
7.28 3.50 3.48 2.44 2.43 2.42 2.14 2.09 1.74 1.59 1.58 1.57 1.55 1.54 1.36 1.34 1.34 1.33 1.32 1.32 1.31 1.30 0.93 0.92 0.91 0.08 0.01 0.01
O
H3C
CH3
12b
2.00 2.91 2.04 2.03 3.02
f1 (ppm)

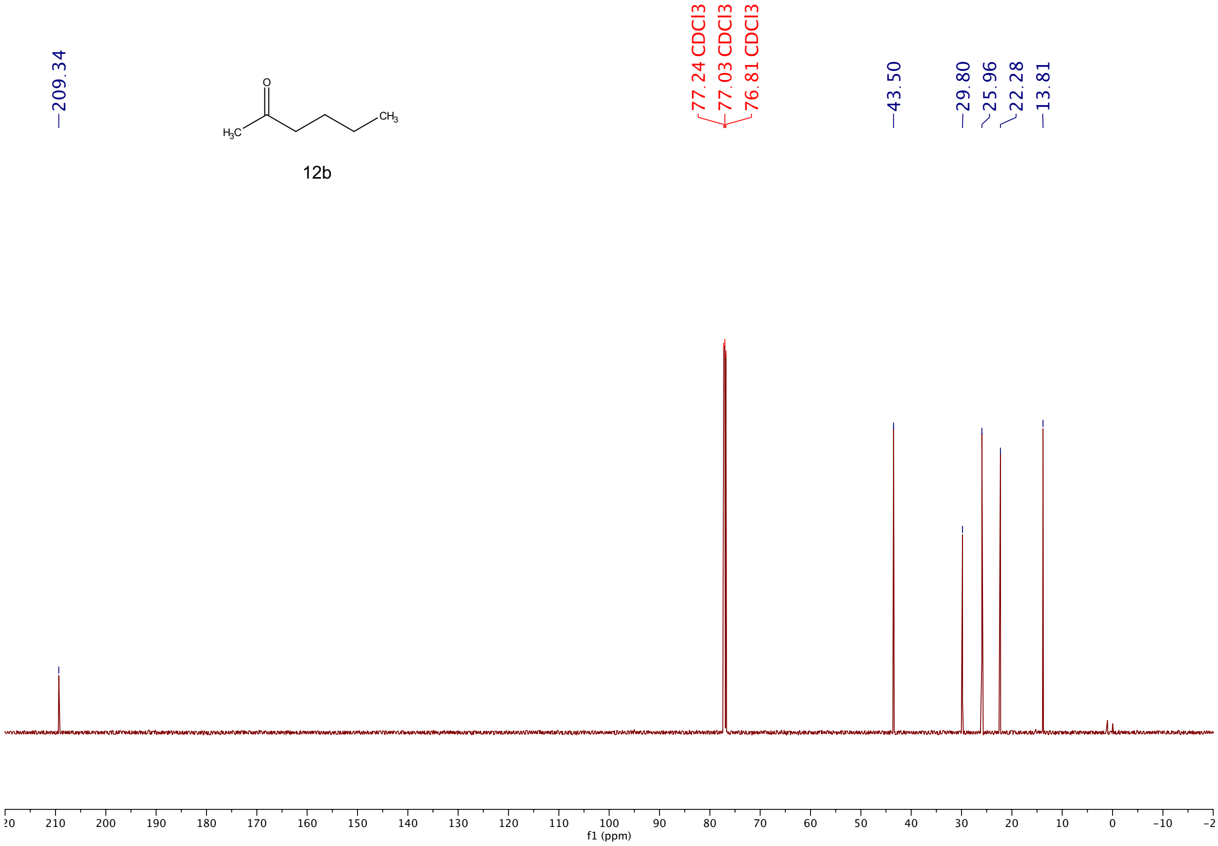
209.34
77.24 CDCl3
77.03 CDCl3
76.81 CDCl3
43.50
29.80 25.96 22.28 13.81
O
H3C
CH3
12b
f1 (ppm)

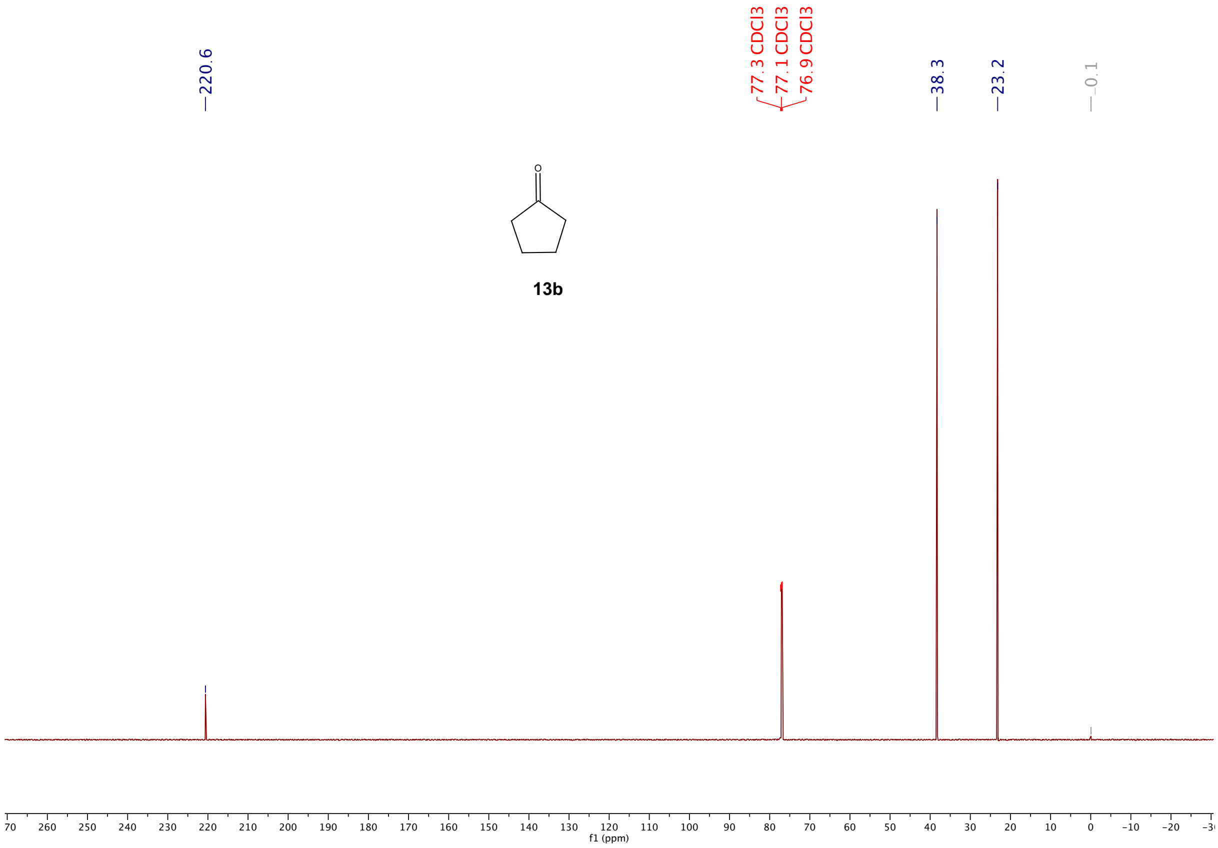
220.6
77.3 CDCl3
77.1 CDCl3
76.9 CDCl3
38.3
23.2
0.1
O
13b
f1 (ppm)

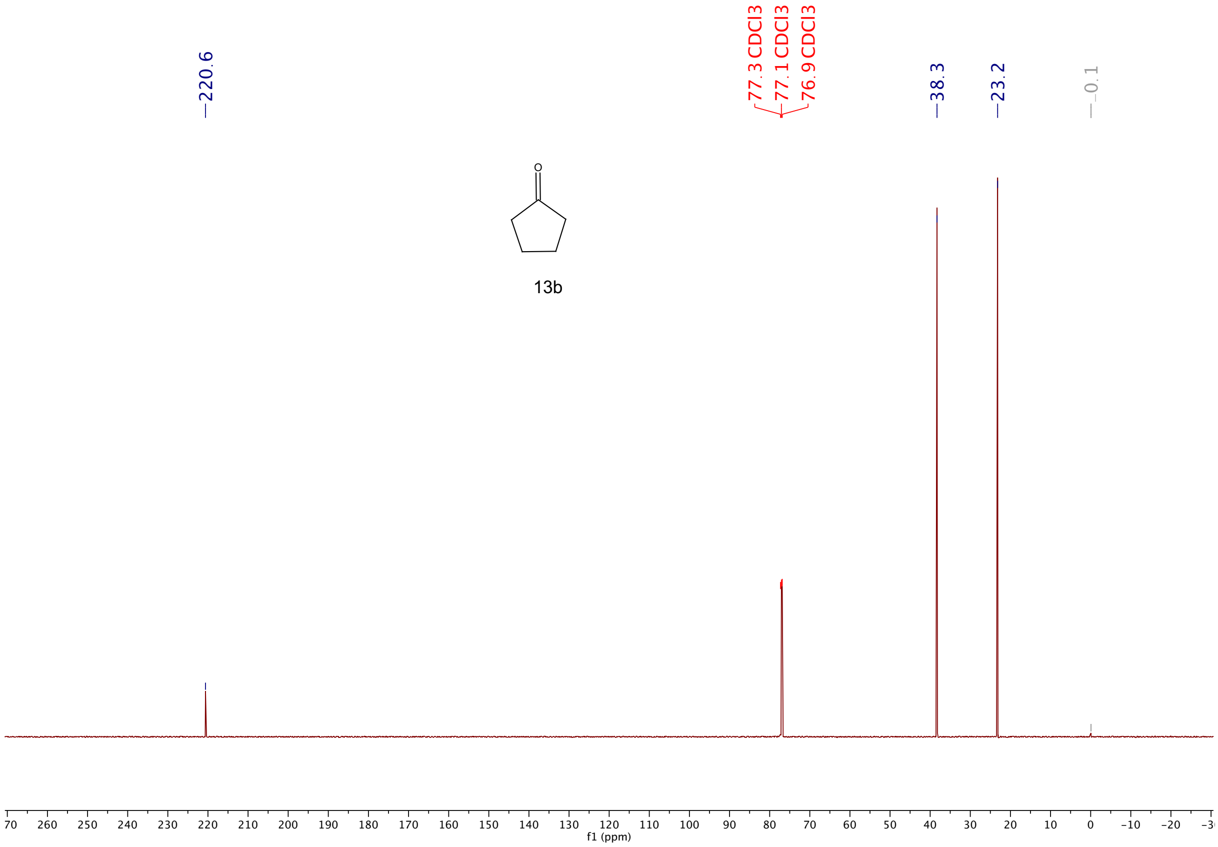
220.6
77.3 CDCl3
77.1 CDCl3
76.9 CDCl3
38.3
23.2
0.1
O
13b
f1 (ppm)

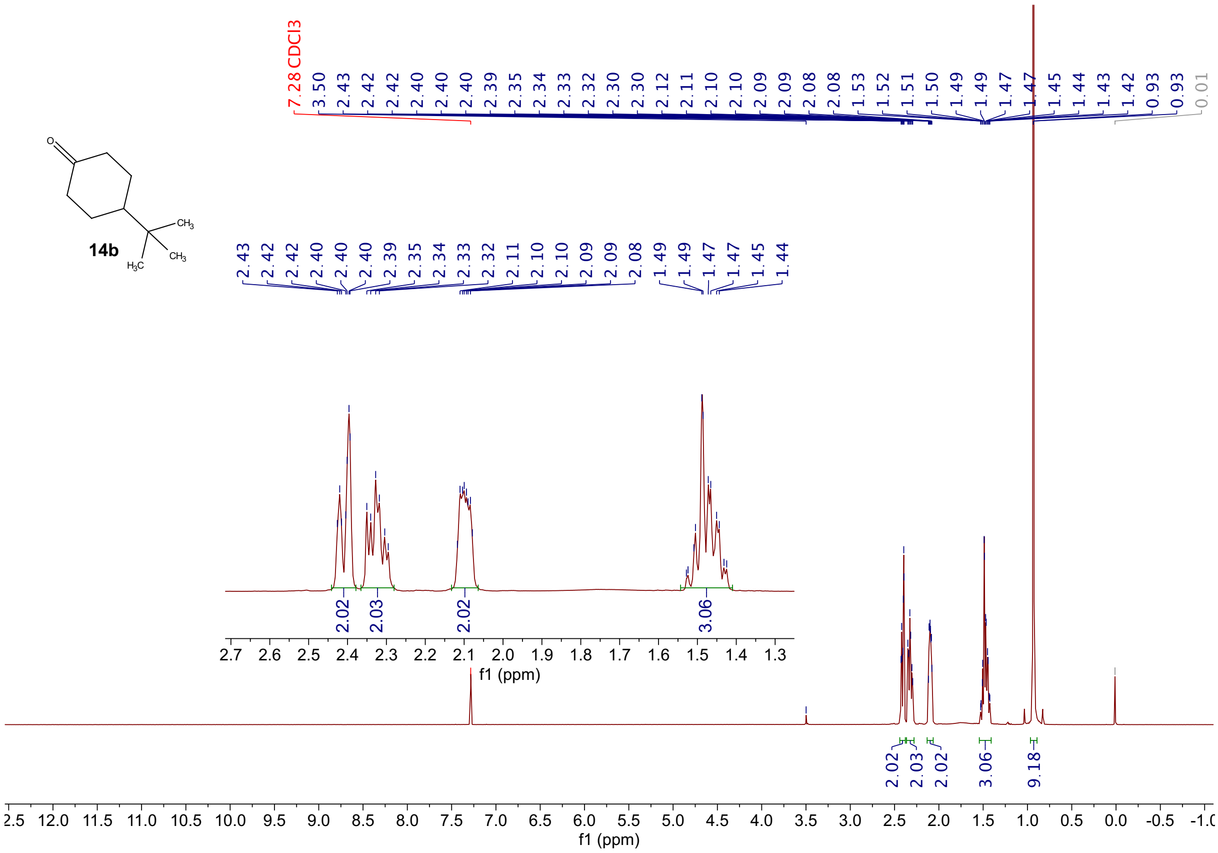
7.28 CDCl3
14b
f1 (ppm)
2.02
2.03
2.02
3.06
9.18

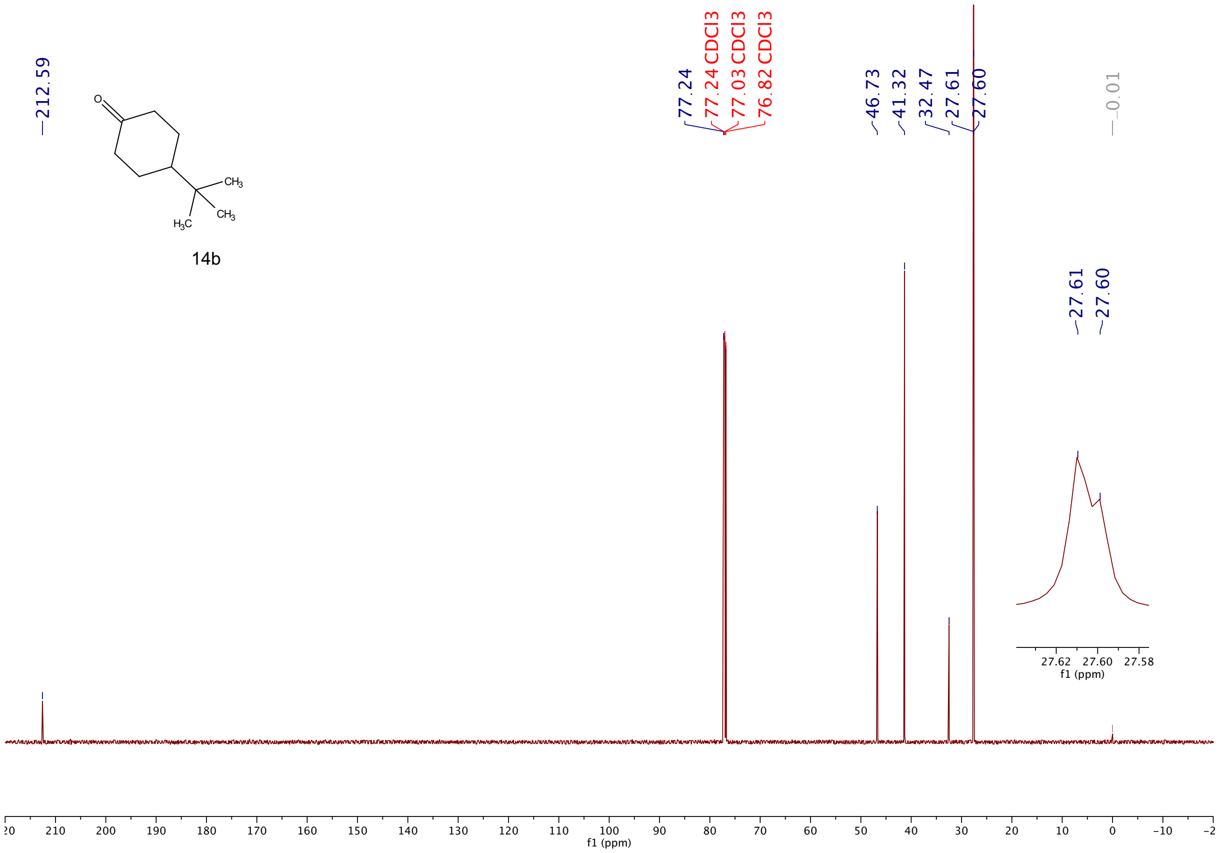
212.59
77.24
77.24 CDCl3
77.03 CDCl3
76.82 CDCl3
46.73
41.32
32.47
27.61
27.60
0.01
14b
f1 (ppm)

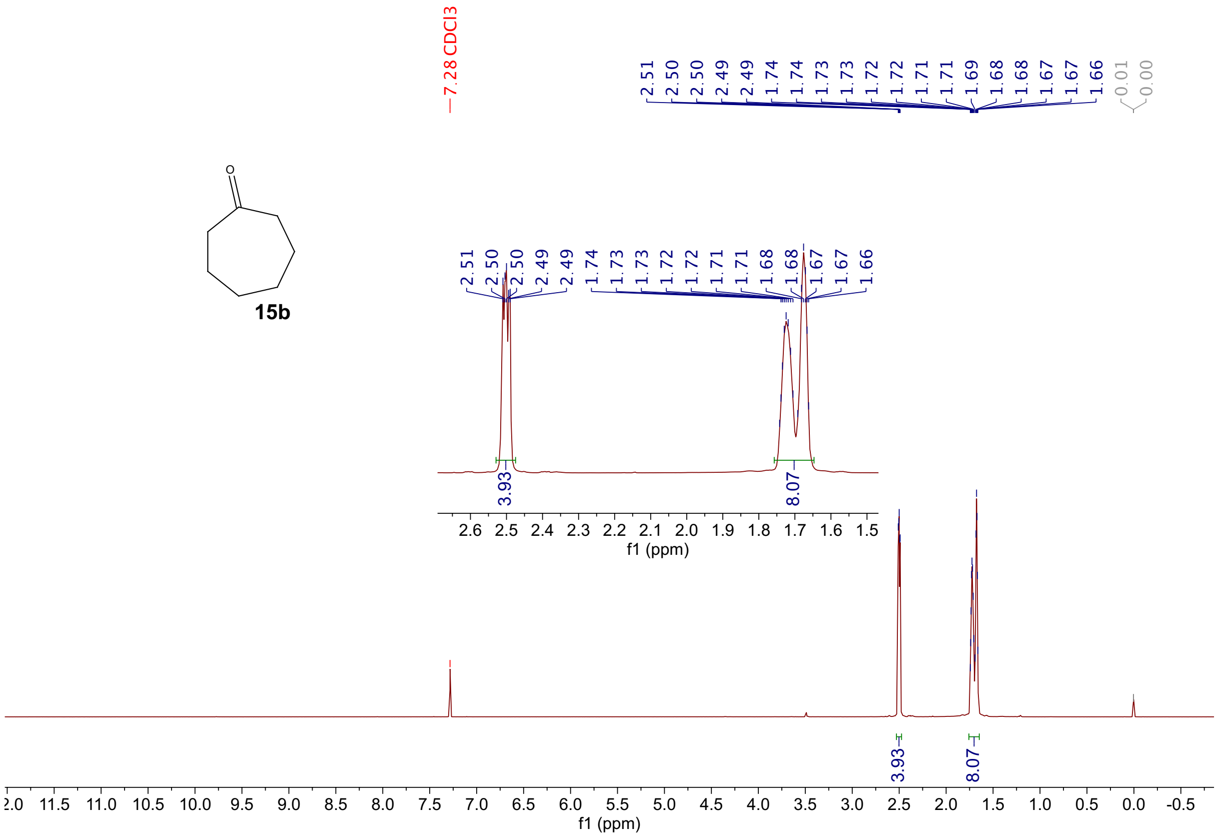
7.28 CDCl3
2.51
2.50
2.50
2.49
2.49
1.74
1.74
1.73
1.73
1.72
1.72
1.71
1.71
1.69
1.68
1.68
1.67
1.67
1.66
0.01
0.00
15b
3.93
8.07
f1 (ppm)

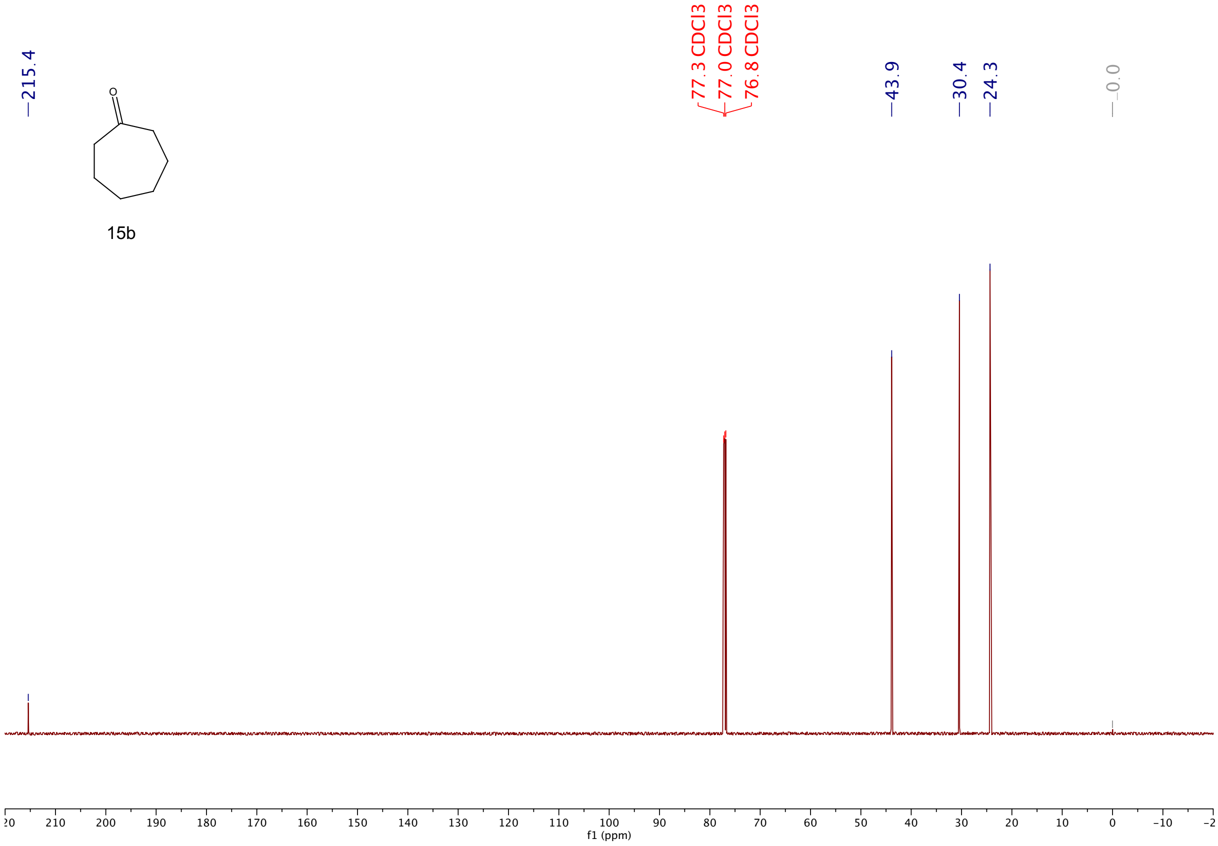
215.4
77.3 CDCl3
77.0 CDCl3
76.8 CDCl3
43.9
30.4
24.3
0.0
15b
f1 (ppm)

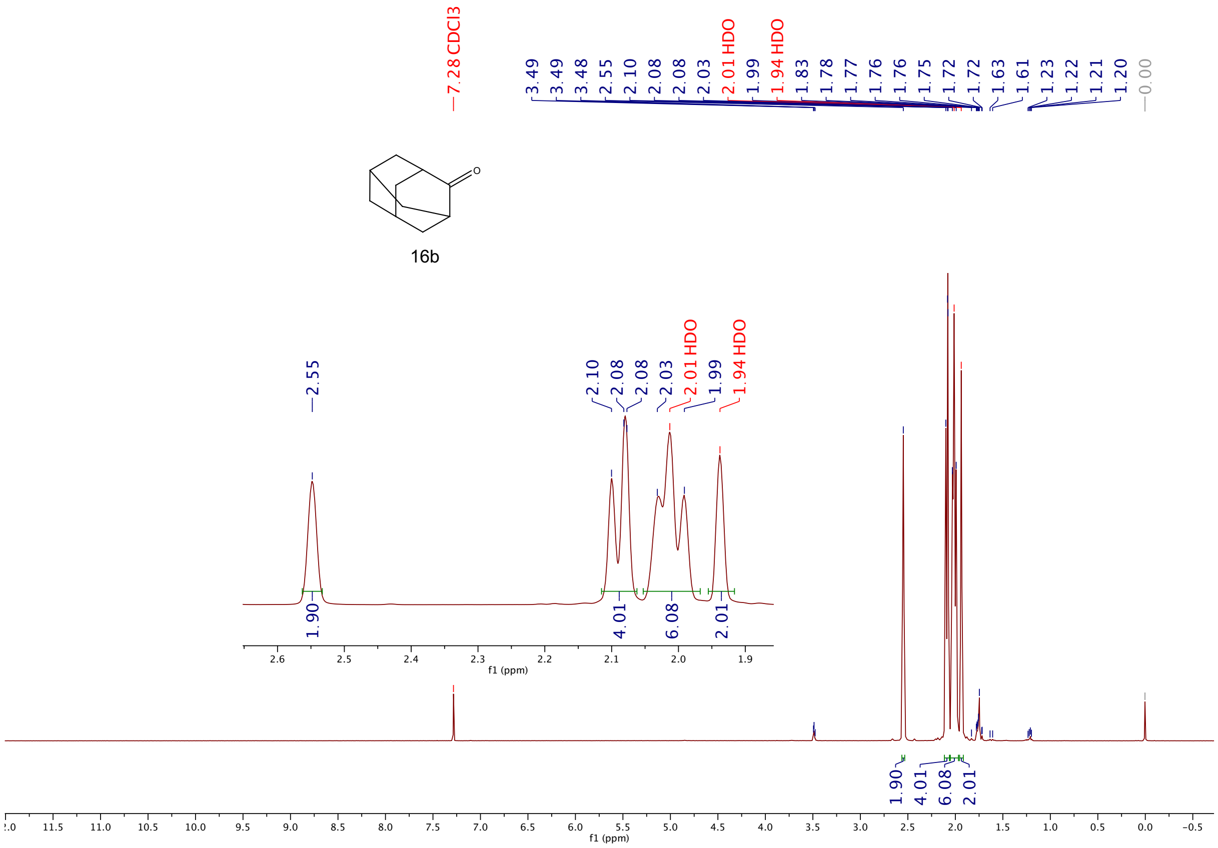
7.28 CDCl3
3.49
3.49
3.48
2.55
2.10
2.08
2.08
2.03
2.01 HDO
1.99
1.94 HDO
1.83
1.78
1.77
1.76
1.76
1.75
1.72
1.72
1.63
1.61
1.23
1.22
1.21
1.20
0.00
16b
O
1.90
4.01
6.08
2.01
f1 (ppm)

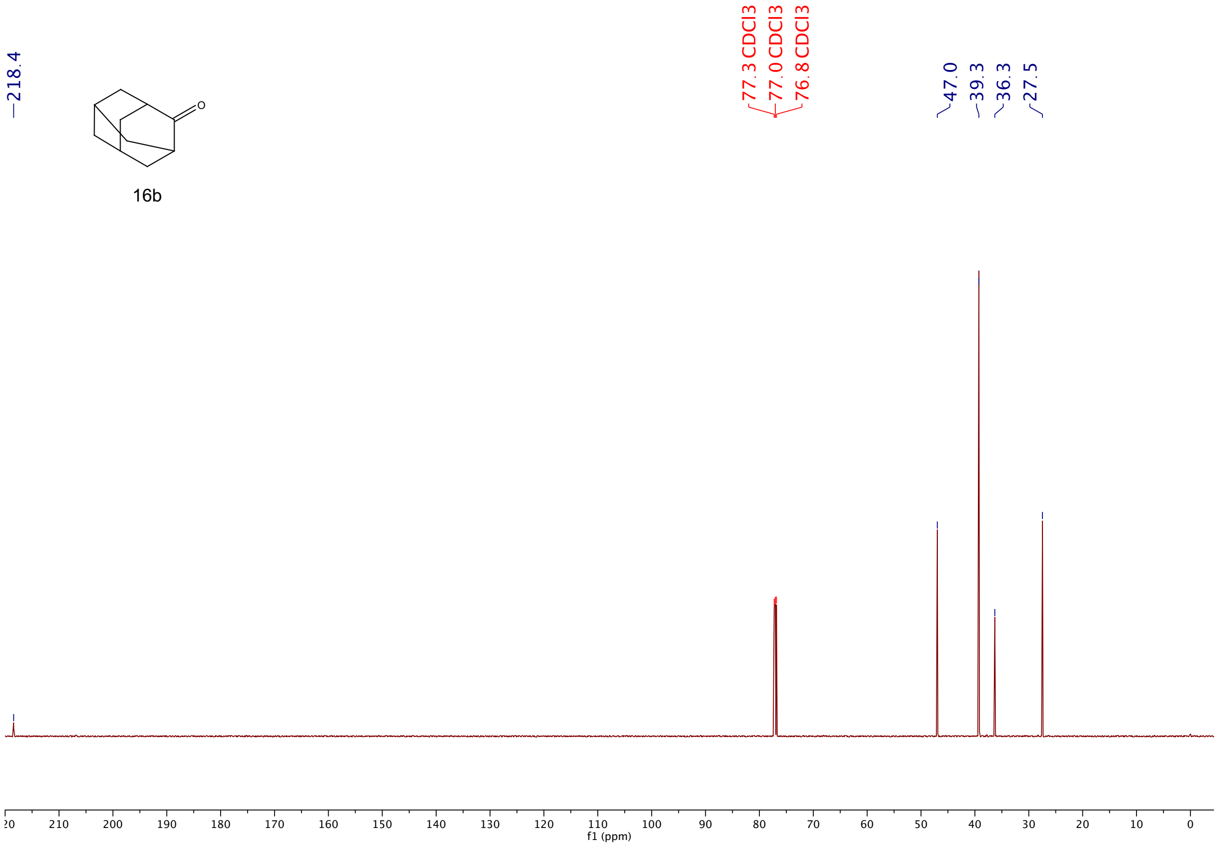
218.4
77.3 CDCl3
77.0 CDCl3
76.8 CDCl3
47.0
39.3
36.3
27.5
16b
O
f1 (ppm)

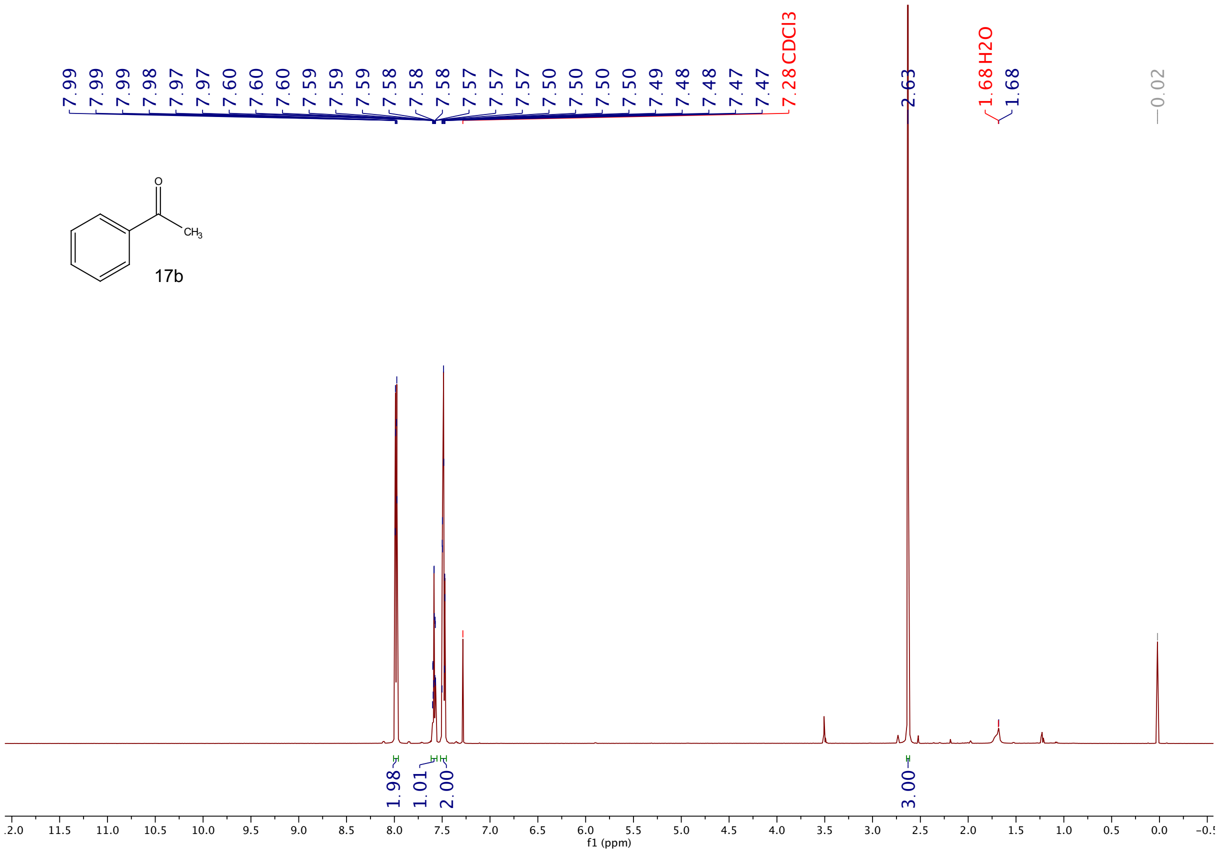
7.99 7.99 7.99 7.98 7.97 7.97 7.60 7.60 7.60 7.59 7.59 7.59 7.58 7.58 7.58 7.57 7.57 7.57 7.50 7.50 7.50 7.50 7.49 7.48 7.48 7.47 7.47
7.28 CDCl3
2.63
1.68 H2O
1.68
-0.02
O
CH3
17b
1.98
1.01
2.00
3.00
f1 (ppm)

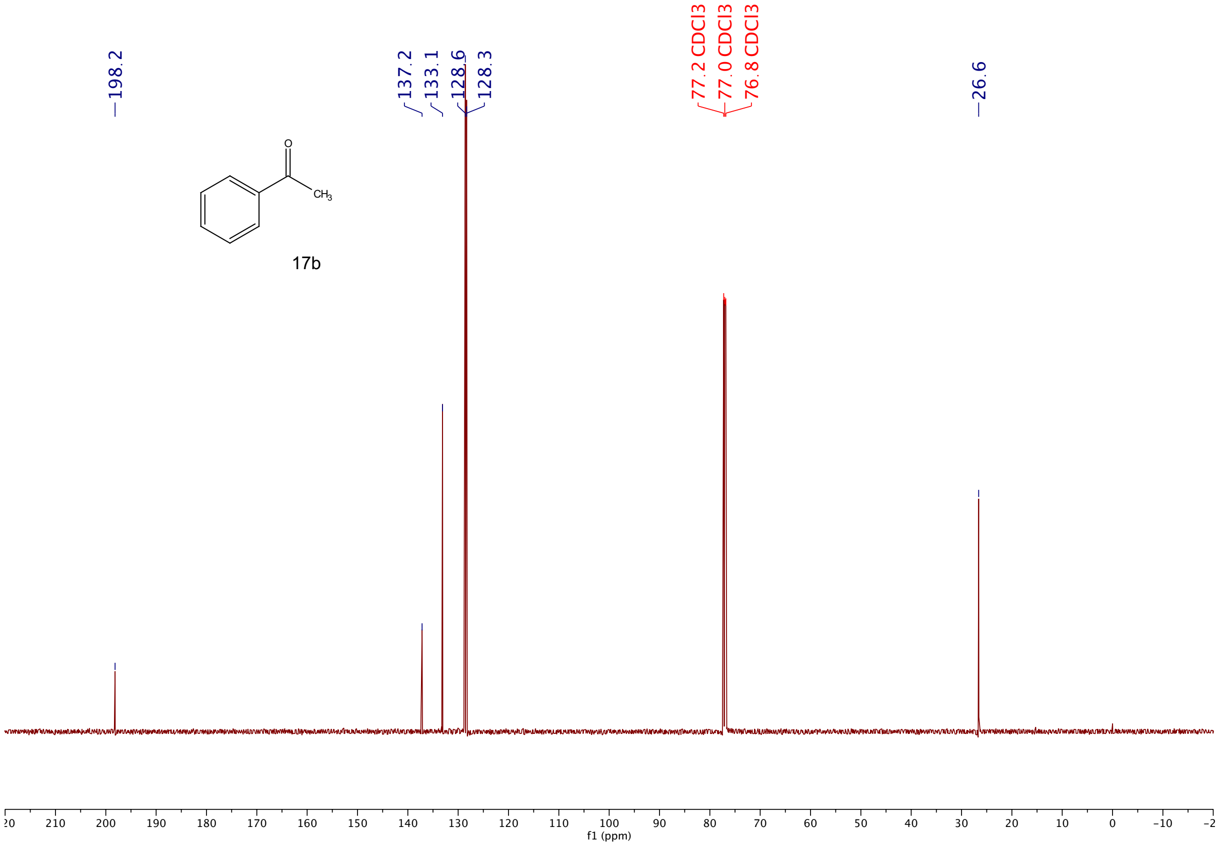
198.2
137.2
133.1
128.6
128.3
77.2 CDCl3
77.0 CDCl3
76.8 CDCl3
26.6
O
CH3
17b
f1 (ppm)

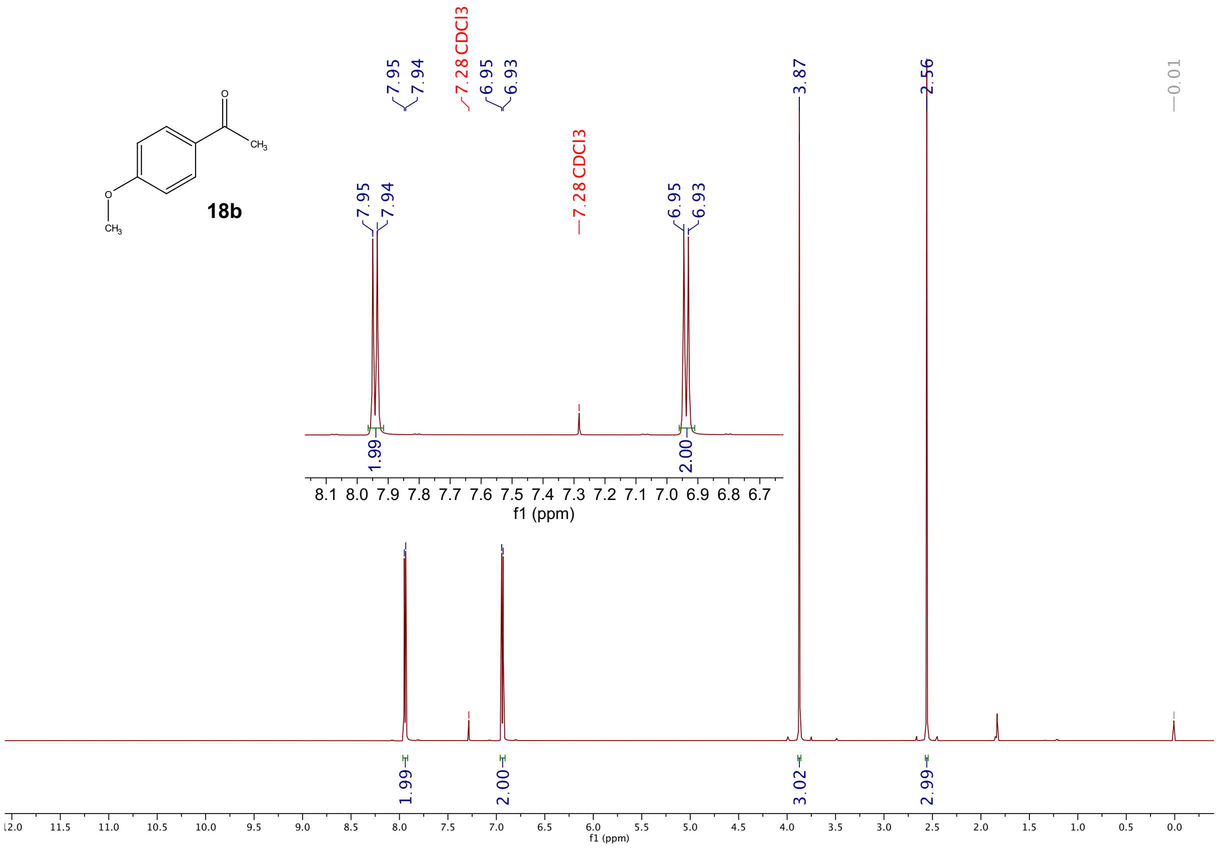
18b
7.95
7.94
7.28 CDCl3
6.95
6.93
3.87
2.56
0.01
1.99
2.00
3.02
2.99
f1 (ppm)

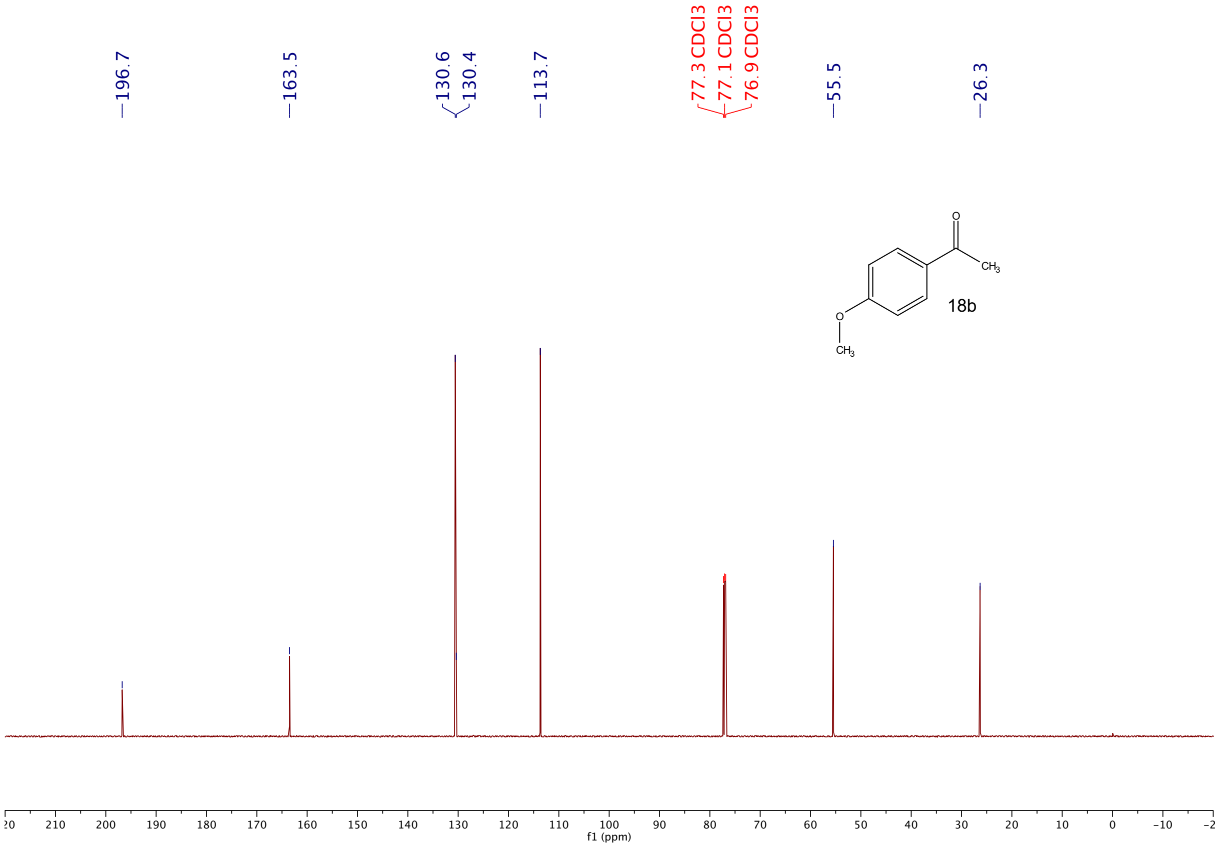
196.7
163.5
130.6
130.4
113.7
77.3 CDCl3
77.1 CDCl3
76.9 CDCl3
55.5
26.3
18b
f1 (ppm)

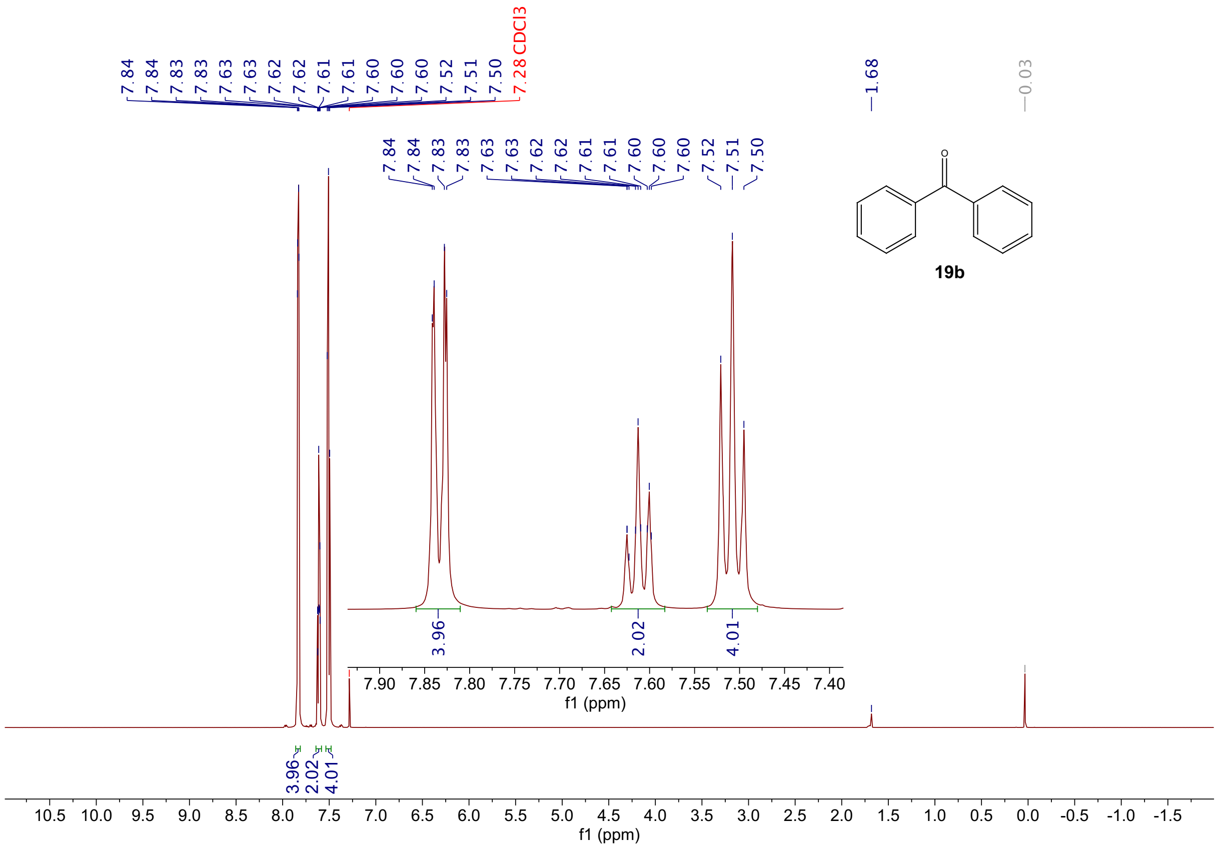
7.84 7.84 7.83 7.83 7.63 7.63 7.62 7.62 7.61 7.61 7.60 7.60 7.60 7.52 7.51 7.50 7.28 CDCl3
1.68
0.03
19b
3.96
2.02
4.01
7.90 7.85 7.80 7.75 7.70 7.65 7.60 7.55 7.50 7.45 7.40
f1 (ppm)
10.5 10.0 9.5 9.0 8.5 8.0 7.5 7.0 6.5 6.0 5.5 5.0 4.5 4.0 3.5 3.0 2.5 2.0 1.5 1.0 0.5 0.0 -0.5 -1.0 -1.5
f1 (ppm)

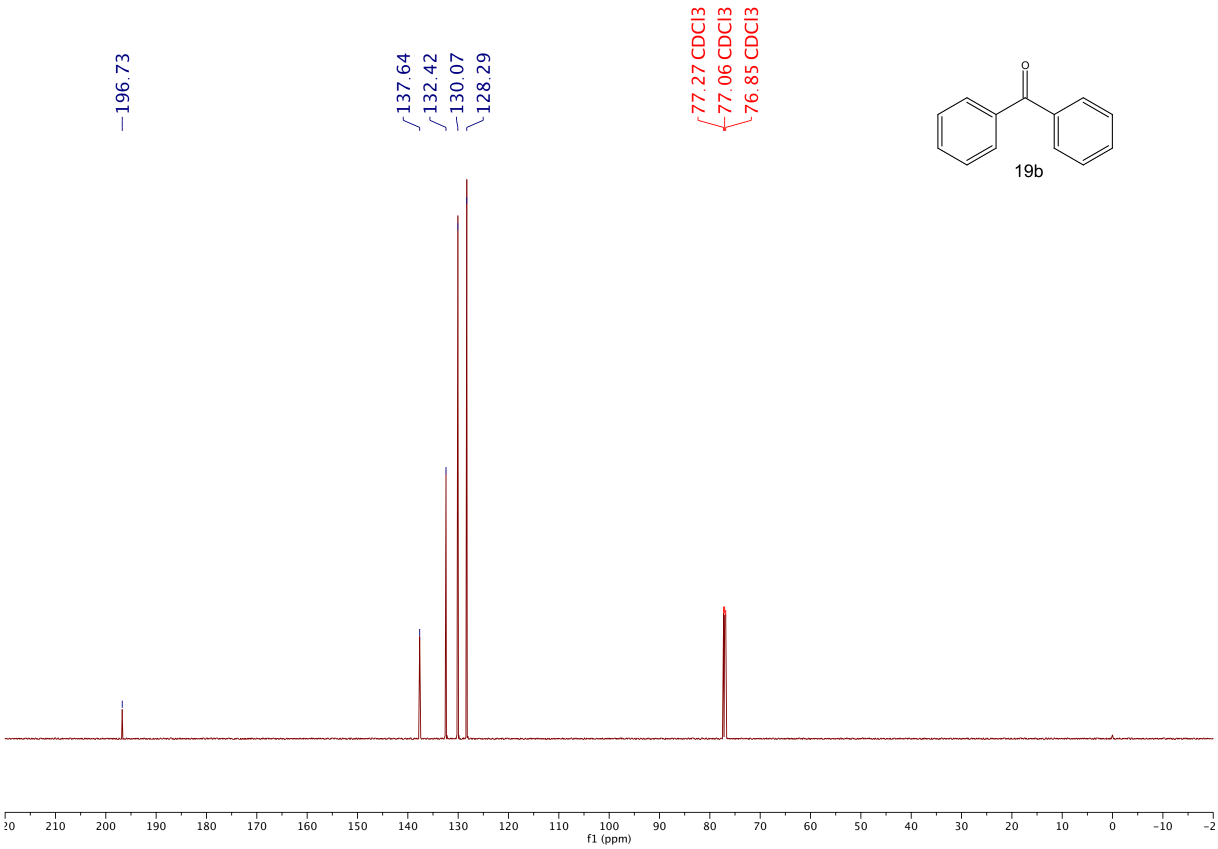
196.73
137.64
132.42
130.07
128.29
77.27 CDCl3
77.06 CDCl3
76.85 CDCl3
19b
210 200 190 180 170 160 150 140 130 120 110 100 90 80 70 60 50 40 30 20 10 0 -10
f1 (ppm)
